# Assessment of Cardiovascular Functioning Among Regular Kratom (*Mitragyna speciosa* Korth) Users: A Case Series

**DOI:** 10.3389/fphar.2021.723567

**Published:** 2021-08-24

**Authors:** Mohammad Farris Iman Leong Bin Abdullah, Darshan Singh

**Affiliations:** ^1^ Lifestyle Science Cluster, Advanced Medical and Dental Institute, Universiti Sains Malaysia, Penang, Malaysia; ^2^ Centre for Drug Research, Universiti Sains Malaysia, Penang, Malaysia

**Keywords:** cardiovascular functioning, electrocardiogram, echocardiogram, serum mitragynine, regular kratom use

## Abstract

Multiorgan toxicities have been extensively reported in kratom (*Mitragyna speciosa* Korth) users in Western countries but not in Southeast Asia. Existing literature argued that this discrepancy may be due to underreporting of kratom-related toxicity cases in Southeast Asia. Hence, this case series filled the research gap by clinically assessing the cardiovascular functioning and serum mitragynine level of regular kratom users in its traditional settings in Malaysia. Nine regular kratom users without history of polysubstance use were recruited from the same community via snowball sampling and were subjected to electrocardiogram (ECG) and echocardiogram assessments. Serum mitragynine analysis was also performed by solid-phase extraction and liquid chromatography-tandem mass spectrometry. The mean serum mitragynine level was 10.3 mg/L (SD = 6.9) and ranged from 2.5 mg/L to 22.4 mg/L. Those who consumed an average daily quantity of four or more glasses of brewed kratom juice (*p* = 0.045) and those who had prolonged QTc intervals (*p* = 0.017) had significantly higher serum mitragynine level. Echocardiographic findings of all the respondents were normal except one reported left ventricular hypertrophy and another had trivial tricuspid regurgitation with pulmonary artery systolic pressure (PASP) of 10 + 5 mmHg. Regular kratom use without concomitant use of other illicit substances may not provoke any risk of cardiovascular impairment or toxicity except for prolonged QTc interval, which appeared to be dose dependent. However, as this study was limited by a small sample size, future studies with larger sample size are warranted to confirm our findings.

## Introduction

The leaves of *Mitragyna speciosa* (Korth) or better known as kratom, a subtropical plant native to the region of Southeast Asia, exhibits psychotropic properties and has been used as a traditional remedy for symptomatic relief of various illnesses. It has been used in Thailand and Malaysia for centuries but for the past decade, its use in Western countries, such as United States and European nations, as a self-prescribe medication for depression, anxiety disorders, chronic pain and as a substitute to illicit and prescription opioids soared exponentially (Grundmann, 2017). Despite its therapeutic potential, kratom was listed as “drug of concern” by the U.S. Drug Enforcement Administration (DEA) in response to multiple reports of toxicity and mortality cases possibly related to kratom use (Fluyau and Revadigar, 2017).

Reports of individual cases of multiorgan toxicities in U.S. and Europe have been published. Several case reports of kratom induced hepatitis, intrahepatic cholestasis, hepatomegaly, and acute liver failure have been documented (Dorman et al., 2015; Griffiths et al., 2018; Waters et al., 2018; Antony and Lee, 2019; Fernandes et al., 2019; Osborne et al., 2019). Post-mortem findings alleged to be associated with accidental deaths have linked kratom use with hepatomegaly, congested liver, fatty liver, liver steatosis, and liver fibrosis (Corkery et al., 2019). The renal toxidrome reportedly link to kratom use are congested kidney, distended bladder, urinary retention, kidney stones, and nephritis (Corkery et al., 2019). Despite various toxidrome reported in the West, toxicity related to kratom use has not been documented in Southeast Asia (Singh et al., 2016).

Cases of cardiotoxicity have also been documented, such as ventricular arrythmia, ventricular tachycardia, ventricular fibrillation, cardiomegaly, cardiomyopathy, coronary atherosclerosis, focal band necrosis in myocardium, myocardial infarction, hypertensive cardiovascular disease, left ventricular hypertrophy, myocardial ischemia, and myocarditis (Aggarwal et al., 2018; Corkery et al., 2019; ELJack et al., 2020; Eastlack et al., 2020; Sheikh et al., 2021). Again, cardiotoxicity has only been reported in the West, but not in Southeast Asia. The current literature has ostensibly suggested that this discrepancy may be caused by the underreporting of kratom-related toxicity cases in Southeast Asia (Corkery et al., 2019). In addition, *in vitro* studies on human induced pluripotent stem cell-derived cardiomyocytes indicated that mitragynine, the most abundant psychoactive alkaloid in kratom extract, is capable of prolonging the action potential duration of cardiomyocytes and increased the risk of prolonged QTc interval and torsades de pointes (Lu et al., 2014). This was followed by a recent study of Electrocardiogram (ECG) in Malaysian subjects which highlighted that regular kratom use may increase the risk of borderline QTc interval [431–450 ms; (Leong Abdullah et al., 2021)]. Given the cardiovascular risk, Leong Abdullah et al. (2021) study was limited by the absence of the serum mitragynine analysis in regular kratom users and cardiac pathology was not examined with echocardiogram. Hence, we conducted this case series to fill the research gap by examining the cardiovascular functioning with electrocardiogram and echocardiogram, and serum mitragynine analysis was performed among regular kratom users in its traditional settings in Malaysia. To the best of our knowledge, to date this case series was the first to examine the echocardiogram and serum mitragynine level in addition to electrocardiogram analysis to assess cardiovascular functioning among regular kratom users without concomitant use of other illicit substances.

## Methods

### Respondent Recruitment

All the nine regular kratom users were recruited from a targeted community located in the state of Penang in Peninsular Malaysia which has a high prevalence of kratom use. Snowball sampling was employed in which an informant who was a regular kratom user who resided in the targeted community was briefed on the purpose and procedures of the case series and assisted in the recruitment drive. The eligibility criteria for the study were: 1) self-reported as a regular kratom user who consumed kratom on a daily basis in the last 12 months, and 2) have no significant history of medical illness, illicit drug and alcohol consumption, psychiatric disorder and had not consumed any medications on regular basis. The eligible subjects were then asked to provide their written informed consent, before they were enrolled in the assessments which were carried out at the Advanced Medical and Dental Institute, Universiti Sains Malaysia. This study has received approval from the Human Ethics Committee of Universiti Sains Malaysia (code: USM/JEPeM/19010054). Respondents were also screened with rapid urine test-kits for opioids, methamphetamine/amphetamine, ketamine, benzodiazepine, cannabis, methadone, and phencyclidine.

### Study Procedures

**FIGURE 1:**
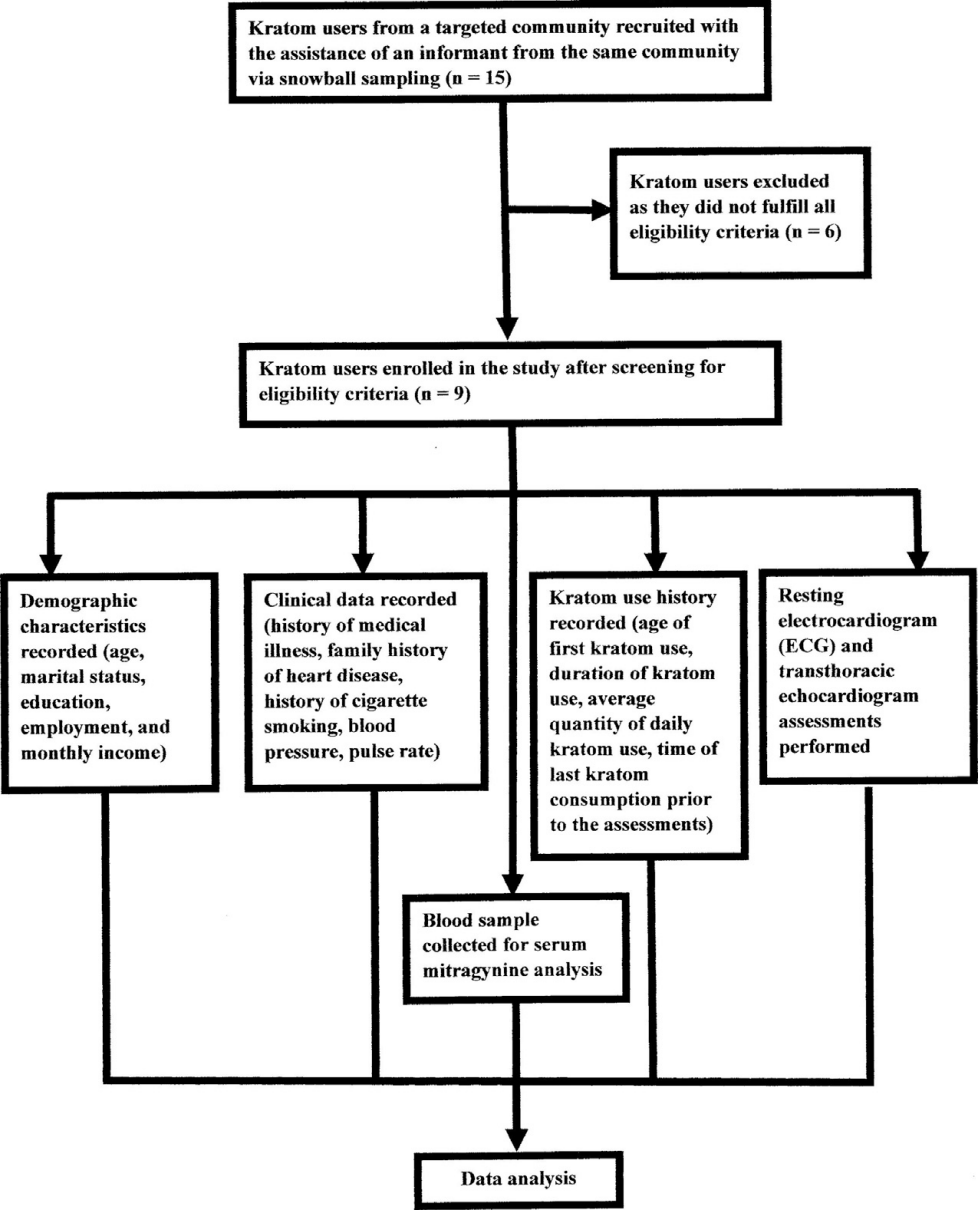
Schematic presentation of the study design.

Data on demographic characteristics, clinical data, and kratom use history were elicited. Then, the resting electrocardiogram (ECG) and transthoracic echocardiogram assessments of all the respondents were carried out. Blood sample was also collected from each respondent for serum mitragynine analysis and to evaluate the physical health status of the respondents. Figure 1 summarizes the schematic presentation of the study design.

#### Demographic and Clinical Characteristics

In the context of demographic characteristics, the data collected include age, marital status, education, employment, and monthly income. Responses for age were reported as continuous variable. Responses for marital status were recorded as either married, single, or divorce/widower. Responses for education were reported as studied up to primary education, studied up to secondary education, or studied up to tertiary education. Responses for employment were grouped into either employed or unemployed. Finally, responses for monthly income were recorded as continuous variable.

As for clinical characteristics of the respondents, the data recorded include history of medical illness, family history of heart disease, history of cigarette smoking, blood pressure and resting pulse rate. These data were assessed and recorded by the medical doctor in the research team. History of medical illness and family history of heart disease were reported as either presence or absence. Since all the respondents had history of cigarette smoking on a daily basis, this variable was assessed based on the question, “On average, how many sticks of cigarette do you smoke in a day?” Response categories ranged from 1 to 30 sticks. Blood pressure was measured in mmHg and as the average of two readings taken for each respondent. In addition, the resting pulse rate of the respondents were measured in beats per minute.

#### Kratom Use History

Kratom use history elicited from the respondents included age of first kratom use, duration of kratom use, average daily quantity of kratom use, and time of last kratom consumption prior to the assessments (blood collection, ECG, and echocardiogram). History of illicit drugs consumption was also recorded. The age of first kratom use was evaluated through the question, “What was your age when you first started to consume kratom?.” Response categories ranged from 6 years old to 40 years old. The duration of kratom use was assessed through the question, “How many years have you been consuming kratom juice?.” Response categories ranged from 1 year to 40 years. Since all the respondents consumed kratom on a daily basis, the average daily quantity of kratom use was elicited through the question, “On average, how many glasses of kratom juice do you consumed in a day?.” The response categories ranged from 1 to 30 glasses per day. The time of last kratom use prior to the assessments was evaluated through the question, “How many hours ago did you last consumed kratom prior to blood collection, ECG and echocardiogram assessments?.” Response categories ranged from 1 to 72 h. History of illicit drugs consumption was assessed through the question, “Do you currently consumed or in the past had consumed any illicit drugs such as heroin, morphine, methamphetamine or Ice/Syabu, Ecstasy, benzodiazepine or Erimin 5, cannabis or ganja, ketamine, LSD, phencyclidine, inhalant, and/or alcohol?.” The responses were reported as either presence or absence.

#### Resting ECG and Echocardiogram Assessments

The resting ECG and echocardiogram assessments, and the interpretation of the findings were carried out by a trained cardiologist. The measurements of the ECG parameters performed in this study are as follow:

1) PR interval was measured from the beginning of the upslope of the P wave to the beginning of the QRS wave. The normal range was from 0.12 to 0.20 s (Rosenthal, 2020).
2) QRS interval was measured from the beginning of the Q wave (at the end of PR interval) to the end of the S wave. The normal range was from 0.08 to 0.10 s (Rosenthal, 2020).
3) QT interval was measured from the beginning of the QRS complex to the end of the T wave (Rosenthal, 2020).
4) RR interval is the distance between the peaks of two consecutive QRS complex. RR interval was measured as 60/heart rate in this study. The normal range was from 0.6 to 1.2 s (Vandenberk et al., 2016; Rosenthal, 2020).

The definitions of abnormal ECG in this study are as follow:

1) Left axis deviation was defined when lead I was positive but lead aVF was negative (0⁰ to −90⁰) according to the quadrant or two-lead approach (Surawicz et al., 2009).
2) Sinus tachycardia was defined if the heart rate was greater than 100 beats per minute with regular heart rhythm and a normal P wave [upright, normal morphology and consistent; (Crawford, 2017)].
3) A normal corrected QT (QTc) interval in male subjects was defined as up to 430 ms, while a QTc interval between 431 and 450 ms was considered as borderline QTc interval, and QTc of above 450 ms was considered abnormal or prolonged (Straus et al., 2006). The QTc interval was calculated using Framingham formula [QTc = QT + 0.154 (1-RR)], which has been reported to give the best rate of correction for QT interval (Vandenberk et al., 2016).

The definitions of abnormal echocardiogram in this study are as follow:

1) Left ventricular ejection fraction (LVEF): The modified Simpson method was used to measure LVEF. LVEF for males is evaluated in the following way (American College of Cardiology, 2020):
  a) Normal: 50–70%, midpoint 60%;
  b) Mild dysfunction: 40–49%, midpoint 45%;
  c) Moderate dysfunction: 30–39%, midpoint 35%;
  d) Severe dysfunction: <30%;
  e) Hyperdynamic EF: >70%
2) LVH was reported if left ventricular mass index threshold was >115 g/m^2^ (Barbieri et al., 2012).
3) Mild tricuspid regurgitation was identified if the Doppler echocardiogram showed the following (Hahn et al., 2019):
  a) A small, narrow, central jet;
  b) Soft or incomplete jet by CW Doppler;
  c) PISA radius of ≤0.5 cm at Nyquist 28 cm/s; and
  d) Right ventricle and atrium of normal size

#### Blood Sample Collection

A total of 10 ml of blood sample was collected from each respondent for serum mitragynine analysis. Blood investigations such as complete blood count, renal profile, serum electrolytes, liver function test, thyroid function test, fasting blood sugar, and fasting lipid profile were also performed; in addition to the history and physical examination elicited by the medical doctor in the research team, to rule out the presence of any medical illnesses or abnormal blood parameters. All assessments (history taking, blood investigations, ECG, transthoracic echocardiogram, and serum mitragynine) were performed on the same assessment day.

### Serum Mitragynine Analysis

Serum mitragynine level of all the respondents were analyzed using the solid-phase extraction and liquid chromatography-tandem mass spectrometry. The description of the serum mitragynine analysis is illustrated in Supplementary Appendix S1 in the Supplementary material.

### Data Analysis

All data were analyzed with Statistical Package for Social Sciences (SPSS) version 26 (SPSS 26; SPSS Inc., Chicago, Illinois, United States). The mean serum mitragynine of the regular kratom users were reported as the serum mitragynine level was normally distributed (normality evaluated with Shapiro-Wilk test which *p*-value of >0.05). The differences in serum mitrgynine level between kratom users who consumed four glasses or more kratom juice/day and less than four glasses of kratom juice/day as well as between those with prolonged QTc interval and normal QTc was assessed with independent t-test [as the dependent variable of serum mitragynine was normally distributed and t-test is valid for small sample size up to between 2 and 5 subjects per group; (de Winter 2013)]. Statistical significance was set at *p* < 0.05.

## Results

Initially, 15 regular kratom users were identified by the informant from the targeted community. However, only 9 kratom users were enrolled in the study as 6 kratom users did not fulfilled all the eligibility criteria (4 users had history of polysubstance use and 2 users had history of medical illnesses). The details of the respondent’s characteristics (such as demographic and clinical characteristics, kratom use history, the ECG and echocardiogram findings, and serum mitragynine level), as well as the association between average quantity of daily kratom consumption, QTc intervals, and serum mitragynine levels of the respondents are presented below.

### Respondent Characteristics

The details of the demographics and clinical characteristics, vital signs, history of illicit drug and alcohol use, and kratom use characteristics of all the respondents are presented in Table 1. The details of the main ECG findings, other ECG parameters, transthoracic echocardiogram findings, and serum mitragynine level of all the respondents are summarized in Table 2. While the full blood investigation findings of the kratom users are presented in Supplementary Table S1 in the Supplementary material. All the blood investigations (complete blood count, renal profile, serum electrolytes, liver function test, thyroid function test, and fasting blood sugar) of the cases were normal except cases 1, 4, and 7 exhibited high serum triglyceride and cases 5 and 6 had high serum LDL. The selected characteristics of the cases are summarized below:

1) Case 1 was a 19-years old male, who had been using kratom for the past 3 years with an average daily kratom consumption of four glasses of kratom juice and his last kratom consumption was 2 h prior to time of assessment. His ECG indicated sinus tachycardia and prolonged QTc interval (468 ms) with a normal QRS interval (80 ms), while the other ECG parameters were normal. His echocardiogram findings were normal with a LVEF of 65%. He recorded a serum mitragynine level of 9.6 mg/L.
2) Case 2 was a 35-years old male, who had been using kratom for the past 14 years with an average daily kratom consumption of two glasses of kratom juice and his last kratom consumption was 2 h prior to time of assessment. His ECG findings were unremarkable where the ECG parameters were all normal. His echocardiogram findings were also normal with a LVEF of 64%. He recorded a serum mitragynine level of 3.6 mg/L.
3) Case 3 was a 23-years old male, who had been using kratom for the past 8 years with an average daily kratom consumption of four glasses of kratom juice and his last kratom consumption was 3 h prior to time of assessment. His ECG indicated prolonged QTc interval (471 ms) with a normal QRS interval (92 ms), while the other ECG parameters were normal. His echocardiogram indicated presence of trivial tricuspid regurgitation with pulmonary artery systolic pressure (PASP) of 10 + 5 mmHg and his LVEF was 61%. He recorded a serum mitragynine level of 11.3 mg/L.
4) Case 4 was a 43-years old male, who had been using kratom for the past 13 years with an average daily kratom consumption of four glasses of kratom juice and his last kratom consumption was 2 h prior to time of assessment. His ECG findings revealed presence of left axis deviation and prolonged QTc interval (466 ms) with a normal QRS interval (84 ms), while the other ECG parameters were normal. His echocardiogram indicated left ventricular hypertrophy with a LVEF of 63%. He recorded a serum mitragynine level of 22.4 mg/L.
5) Case 5 was an 18-years old male, who had been using kratom for the past 5 years with an average daily kratom consumption of three glasses of kratom juice and his last kratom consumption was 2 h prior to time of assessment. His ECG findings were unremarkable except for presence of sinus tachycardia. His echocardiogram was normal with a LVEF of 67%. He recorded a serum mitragynine level of 6.8 mg/L.
6) Case 6 was a 21-years old male, who had been using kratom for the past 14 years with an average daily kratom consumption of four glasses of kratom juice and his last kratom consumption was 2 h prior to time of assessment. His ECG findings were unremarkable where the ECG parameters were all normal. His echocardiogram findings were also normal with a LVEF of 63%. He recorded a serum mitragynine level of 8.0 mg/L.
7) Case 7 was an 18-years old male, who had been using kratom for the past 4 years with an average daily kratom consumption of four glasses of kratom juice and his last kratom consumption was 3 h prior to time of assessment. His ECG findings revealed presence of prolonged QTc interval (467 ms) with a normal QRS interval of 100 ms, while the other ECG parameters were normal. His echocardiogram indicated left ventricular hypertrophy with a LVEF of 68%. He recorded a serum mitragynine level of 20.4 mg/L.
8) Case 8 was a 22-years old male, who had been using kratom for the past 8 years with an average daily kratom consumption of 6 glasses of kratom juice and his last kratom consumption was 2 h prior to time of assessment. His ECG findings were unremarkable where the ECG parameters were all normal. His echocardiogram findings were also normal with a LVEF of 65%. He recorded a serum mitragynine level of 8.5 mg/L.
9) Case 9 was an 18-years old male, who had been using kratom for the past 2 years with an average daily kratom consumption of three glasses of kratom juice and his last kratom consumption was 2 h prior to time of assessment. His ECG findings were unremarkable where all the ECG parameters were all normal. His echocardiogram findings were also normal with a LVEF of 69%. He recorded a serum mitragynine level of 2.5 mg/L.

**TABLE 1:** Detailed demographic and clinical data, and kratom use characteristics of the respondents.

Variables	Case 1	Case 2	Case 3	Case 4	Case 5	Case 6	Case 7	Case 8	Case 9
Age	19 years	35 years	23 years	43 years	18 years	21 years	18 years	22 years	18 years
Marital status	Single	Single	Single	Married	Single	Single	Single	Single	Single
Education	Up to secondary education	Up to secondary education	Up to secondary education	Up to secondary education	Up to secondary education	Up to tertiary education	Up to secondary education	Up to secondary education	Up to secondary education
Employment	Unemployed	Employed	Employed	Employed	Employed	Unemployed	Employed	Unemployed	Unemployed
Monthly income	<RM 1000	RM 1800	<RM 1000	RM 2500	RM 1100	<RM 1000	RM 1200	<RM 1000	RM 1400
History of medical illness	No	No	No	No	No	No	No	No	No
Family history of heart diseases	No	No	No	No	No	No	No	No	No
Average quantity of daily cigarette smoking (sticks/day)	20	20	20	20	3	10	20	20	5
Blood pressure (mmHg)	133/91	116/68	136/63	157/94	127/82	133/74	110/65	128/77	135/76
Pulse rate (beats/minute)	120	73	117	89	117	81	74	96	86
Age started using kratom	16 years old	21 years old	13 years old	30 years old	13 years old	21 years old	14 years old	14 years old	16 years old
Duration of kratom use	3 years	14 years	8 years	13 years	5 years	14 years	4 years	8 years	2 years
Average daily quantity of kratom use (glasses of kratom juice per day)	4	2	4	4	3	2	4	6	3
Time of last kratom consumption prior to the assessments	2 h prior	2 h prior	3 h prior	2 h prior	2 h prior	2 h prior	3 h prior	2 h prior	2 h prior
History of intake of other illicit drugs and alcohol	No	No	No	No	No	No	No	No	No

**TABLE 2:** Cardiovascular findings and serum mitragynine level of the respondents.

case	Main ECG findings	Other ECG parameters	Echocardiogram findings	Serum mitragynine (mg/L)
Case 1	-Sinus tachycardia	-PR interval = 120 ms	-All chamber size normal	9.6
	-Normal axis		-LVEF = 65%	
	-No ischemic changes	- QRS interval = 80 ms	-No MR by CFM	
	-No heart block		-No AR/AS by CFM	
	-QTc = 468 ms	-QT interval = 393 ms	-No TR by CFM	
			-No PR by CFM	
		-RR interval = 512 ms	-No pericardial effusion	
			-No RWMA	
			-No intracardiac shunt	
Case 2	-Sinus rhythm	-PR interval = 180 ms	-All chamber size normal	3.6
	-Normal axis		-LVEF = 64%	
	-No ischemic changes	- QRS interval = 100 ms	-No MR by CFM	
	-No heart block		-No AR/AS by CFM	
	-QTc = 428 ms	-QT interval = 426 ms	-No TR by CFM	
			-No PR by CFM	
		-RR interval = 984 ms	-No pericardial effusion	
			-No RWMA	
			-No intracardiac shunt	
Case 3	-Sinus rhythm	-PR interval = 170 ms	-All chamber size normal	11.3
	-Normal axis		-LVEF = 61%	
	-No ischemic changes	-QRS interval = 92 ms	-No MR by CFM	
	-No heart block		-No AR/AS by CFM	
	-QTc = 472 ms	-QT interval = 432 ms	-Trivial TR by CFM with PASP 10 + 5 mmHg	
			-No PR by CFM	
		-RR interval = 741 ms	-No pericardial effusion	
			-No RWMA	
			-No intracardiac shunt	
Case 4	-Sinus rhythm	-PR interval = 170 ms	-Left ventricular hypertrophy	22.4
	-Left axis deviation		-All chamber size normal	
	-No ischemic changes	-QRS interval = 84 ms	-LVEF = 63%	
	-No heart block		-No MR by CFM	
	-QTc = 466 ms	-QT interval = 415 ms	-No AR/AS by CFM	
			-No TR by CFM	
		-RR interval = 667 ms	-No PR by CFM	
			-No pericardial effusion	
			-No RWMA	
			-No intracardiac shunt	
Case 5	-Sinus tachycardia	-PR interval = 166 ms	-All chamber size normal	6.8
	-Normal axis		-LVEF = 67%	
	-No ischemic changes	-QRS interval = 81 ms	-No MR by CFM	
	-No heart block		-No AR/AS by CFM	
	-QTc = 411 ms	-QT interval = 350 ms	-No TR by CFM	
			-No PR by CFM	
		-RR interval = 600 ms	-No pericardial effusion	
			-No RWMA	
			-No intracardiac shunt	
Case 6	-Sinus rhythm	-PR interval = 137 ms	-All chamber size normal	8.0
	-Normal axis		-LVEF = 63%	
	-No ischemic changes	-QRS interval = 88 ms	-No MR by CFM	
	-No heart block		-No AR/AS by CFM	
	-QTc = 411 ms	-QT interval = 384 ms	-No TR by CFM	
			-No PR by CFM	
		-RR interval = 822 ms	-No pericardial effusion	
			-No RWMA	
			-No intracardiac shunt	
Case 7	-Sinus rhythm	-PR interval = 148 ms	-All chamber size normal	20.4
	-Normal axis		-LVEF = 68%	
	-T inversion over inferior leads (III, aVF)	-QRS interval = 100 ms	-No MR by CFM	
	-No heart block		-No AR/AS by CFM	
	-QTc = 467 ms	-QT interval = 426 ms	-No TR by CFM	
			-No PR by CFM	
		-RR interval = 731 ms	-No pericardial effusion	
			-No RWMA	
			-No intracardiac shunt	
Case 8	-Sinus rhythm	-PR interval = 150 ms	-All chamber size normal	8.5
	-Normal axis		-LVEF = 65%	
	-No ischemic changes	-QRS interval = 100 ms	-No MR by CFM	
	-No heart block		-No AR/AS by CFM	
	-QTc = 424 ms	-QT interval = 374 ms	-No TR by CFM	
			-No PR by CFM	
		-RR interval = 674 ms	-No pericardial effusion	
			-No RWMA	
			-No intracardiac shunt	
Case 9	-Sinus rhythm	-PR interval = 150 ms	-All chamber size normal	2.5
	-Normal axis		-LVEF = 69%	
	-No ischemic changes	-QRS interval = 90 ms	-No MR by CFM	
	-No heart block		-No AR/AS by CFM	
	-QTc = 414 ms	-QT interval = 352 ms	-No TR by CFM	
			-No PR by CFM	
		-RR interval = 600 ms	-No pericardial effusion	
			-No RWMA	
			-No intracardiac shunt	

LVEF, left ventricular ejection fraction; CFM, colour flow mapping; RWMA, regional wall motion abnormalities; MR, mitral regurgitation; AR, aortic regurgitation; AS, aortic stenosis; TR, tricuspid regurgitation; PR, pulmonary regurgitation.

### The Association Between Average Quantity of Daily Kratom Use, QTc Intervals, and Serum Level of Mitragynine Among the Regular Kratom Users

The association between average daily quantity of kratom use, QTc intervals, and serum mitragynine levels of the respondents are summarized in Table 3. Regular kratom users who consumed an average daily quantity of four or more glasses of kratom juice (freshly brewed kratom juice) registered a significantly higher serum mitragynine level compared with those who consumed less than four glasses of kratom juice [mean serum mitragynine _(< 4 glasses)_ = 4.300, standard deviation (SD) = 2.234; mean serum mitragynine _(≥ 4 glasses)_ = 13.367, SD = 6.356; *p* = 0.045]. Similarly, respondents who recorded prolonged QTc interval corresponded to significantly higher serum mitragynine level compared with those who had normal QTc interval [mean serum mitragynine _(normal QTc)_ = 5.880, SD = 2.685; mean serum mitragynine _(prolonged QTc)_ = 15.925, SD = 6.412; *p* = 0.017].

**TABLE 3:** The association between average daily quantity of kratom use, QTc intervals, and serum level of mitragynine among the regular kratom users.

Variables	Mean serum mitragynine (SD)	Mean difference	t	p-value
Average daily quantity of kratom use				
<4 glasses (*n*= 3)	4.300 (2.234)	−9.067	−3.129	0.017^a^
≥4 glasses (*n* = 6)	13.367 (6.356)	—	—	—
QTc intervals				
Normal (<430 ms) (*n* = 5)	5.880 (2.685)	−10.045	−2.934	0.045^a^
Prolonged (>50 ms) (*n* = 4)	15.925 (6.412)	—	—	—

a Statistical significance at *p* < 0.05, SD, standard deviation.

## Discussion

This case series examined the cardiovascular functioning and serum mitragynine level of regular kratom users who ingested brewed kratom juice. Our findings pinpointed to a few salient points among the case series of regular kratom users: 1) the mean serum mitragynine level of all the kratom users was 10.3 mg/L (SD = 6.9) and ranged from 2.5 mg/L to 22.4 mg/L; 2) Higher average daily quantity of kratom use (more than four glasses of kratom juice) was associated with higher serum mitragynine level; 3) 4 cases with serum mitragynine level of ≥9.6 mg/L exhibited prolonged QTc intervals; 4) kratom users with prolonged QTc intervals reported significantly higher serum mitragynine levels compared with those with normal QTc intervals; and 5) echocardiogram and other ECG findings (including the PR interval, QRS interval, and RR interval) were normal for the respondents except left ventricular hypertrophy was reported in one user, T wave inversion in inferior leads (III, aVF) in one user, and trivial tricuspid regurgitation with PASP 10 + 5 mmHg in another user were reported; with serum mitragynine of 22.4, 20.4 and 11.3 mg/L, respectively.

In the context of regular kratom consumption on cardiovascular function, a higher serum mitragynine level was associated with prolonged QTc interval. Our finding is in agreement with a former *in vitro* study of mitragynine effect on human induced pluripotent stem cell-derived cardiomyocytes (hiPSC-CMs; Lu et al., 2014). The QTc interval prolongation was most likely caused by prolonged repolarization and not depolarization since no changes in the QRS complex was found. Mitragynine and its analogues suppressed rapid delayed rectifier potassium current (I_Kr_) by 67–84%, and significantly prolonged action potential duration (APD) in hiPSC-CMs in a dose-dependent manner without exerting changes in the L-type Ca^2+^ current (I_Ca,L_). Hence, mitragynine could induced prolonged QTc interval at increasing serum level (Lu et al., 2014). The human ether-a-go-go-related gene (hERG) encode for a pore forming subunit of the I_Kr_ channel and hence, it is involved in the channel trafficking of ions across the cell membrane of cardiomyocytes. A more recent *in vitro* study on the mechanism of mitragynine-induced inhibition on the human ether-a-go-go-related gene 1a/1b (hERG1a/1b) confirmed that mitragynine suppressed the I_Kr_ current at a half-maximal inhibitory concentration (IC_50_) value of 332.70 nM and induced significant decreased of the fully glycosylated (fg) hERG1a protein expression at lower dose, indicating that mitragynine directly block channel trafficking at lower dosage. Contrastingly, mitragynine at high dose upregulates the core-glycosylated (cg) hERG1a protein expression and hERG1a-Hsp90 complexes, revealing that mitragynine may induced hERG1a channel misfolding and triggered the unfolded protein response (UPR) and endoplasmic reticulum-associated protein degradation (ERAD) system as part of the compensatory mechanism of increasing ER stress (Tay et al., 2019). This possibility warrants further investigation in the future.

A summary of 156 cases of kratom-related deaths in the West and its post-mortem findings revealed that left ventricular hypertrophy was observed in six cases, while myocardial ischemia and infarction were detected in three cases (Corkery et al., 2019). Despite one respondent presented with left ventricular hypertrophy, one respondent had T wave inversion in inferior leads III and aVF (possibly indicative of myocardial ischemia), and another had trivial tricuspid regurgitation. Unfortunately, the small sample size of this case series precludes firm conclusions about the clinical relevance of these findings but does stress the need for further research in larger sample of kratom users to compare with control subjects.

As for the serum mitragynine level which was associated with kratom-related deaths, post-mortem reports of mortality cases affirmed that the mean serum mitragynine level for mortality cases which involved co-administration of kratom with other substances was at 0.890 mg/L (range = 0.000089–16.00 mg/L), while the mean serum mitragynine level for death cases related to kratom use as the sole substance was at 2.128 mg/L (range = 0.016–16.000; Corkery et al., 2019). Interestingly, the mean serum mitragynine reported in this case series was much higher (10.3 mg/L, range = from 2.5 to 22.4 mg/L); but the respondents were only using kratom without the use of other illicit substances, had no history of medical illness, alcohol consumption and psychiatric disorder, and had not consumed any medications on regular basis. As pointed out by Corkery et al. (2019), kratom toxicity in the West may arise from the potentiation effect of mitragynine and its metabolite 7-hydroxymitragynine on other co-administered substances, increasing the latter toxic effects on different organ systems. Mitragynine may also act as CYP2D6 inhibitor, which inhibit the metabolism of co-administered substances in the liver, increasing their toxic potential (Hanapi et al., 2013; Hughes, 2019). Moreover, it is unclear whether the mortality cases reported by Corkery et al. (2019) were caused by kratom use *per se* or have been compounded partially by underlying medical disorders as the health background of the reported death cases were not assessed thoroughly. Besides, kratom users in the United States consists of naïve users and they may not be using kratom on daily basis and experienced tolerance. Hence, they may be more prone to kratom toxicity.

Our findings must be interpreted with caution considering several limitations. First, the sample size of this study was small. Besides, the association between serum mitragynine level and QTc intervals among the respondents which was assessed by univariate analysis may not indicate the causative effect of serum mitragynine level on the QTc intervals. Hence, prospective study with larger sample size and use of more robust statistical analysis is needed to confirm our findings. Second, the case series design may limit the reliability of this study as there is no control group for comparison. Assessing the dose response relationship with the average number of glasses of kratom juice consumed daily may not be optimal as those who consumed higher average daily quantity of kratom may represent two different populations of kratom users, such as those who consumed higher dose of kratom with the absence of tolerance on one hand and those who consumed higher dose of kratom due to extensive tolerance on the other hand. Third, the kratom users were recruited only from one state in Peninsular Malaysia (Penang) and this affects the generalizability of our findings. Besides Penang, kratom use is also common in the states of Perlis and Kedah in Peninsular Malaysia. Fourth, we failed to recruit female kratom users for this case series. Nevertheless, regular female kratom users are rare in Malaysia as most consumed it for its medicinal properties in relieving diarrhea, cough, myalgia, and abdominal discomfort (Singh et al., 2016). Finally, all the respondents in this study consumed kratom on daily basis for longer than 1 year in duration. Hence, the effect of initial or periodic kratom use on the cardiovascular functioning could not be determined in this study. Moreover, since this study excluded kratom users with chronic diseases, the effect of regular kratom use on the cardiovascular functioning of users with comorbid chronic illnesses could not be evaluated.

## Conclusion

To conclude, this was the first case series which investigated cardiovascular functioning and its association with serum mitragynine level among regular kratom users who ingest freshly brewed kratom solution on a daily basis. Our findings add to the paucity of information on kratom side-effects and serves as a guideline to facilitate clinicians to understand that: 1) higher quantity of daily kratom consumption did increase the serum mitragynine level and 2) regular kratom use without concomitant use of other substances (even if the serum mitragynine level of as high as 22.4 mg/L) may not lead to any risk of cardiotoxicity except for prolonged QTc interval, which was dose dependent. However, torsades de pointes was not observed in all the regular kratom users in this study. Hence, kratom users who visited the emergency department suspected of kratom overdose or toxicity warrant an ECG examination, and perhaps Holter monitoring should also be considered. Based on our study findings, in order to delineate kratom’s safety profile, there is an urgent need for studies to assess the serum cardiac markers, echocardiogram, Holter monitoring, serum mitragynine and 7-hydroxymitragynine to fully determine the potential cardiotoxicity risk of regular kratom consumption.

## Data Availability

The raw data supporting the conclusion of this article will be made available by the authors, without undue reservation.

## Supplementary Material

Supplementary Appendix S1 Analysis of Mitragynine in Blood Samples using Liquid Chromatography-Tandem Mass Spectrometry (LC-MS/MS).

## References

[B1] Aggarwal G. Robertson E. McKinlay J. Walter E. (2018). Death from Kratom Toxicity and the Possible Role of Intralipid. J. Intensive Care Soc. 19, 61–63. 10.1177/1751143717712652 29456604 PMC5810870

[B2] American College of Cardiology (2020). Left Ventricular Ejection Fraction LVEF Assessment (Outpatient Setting). Available from: https://www.acc.org/tools-and-practice-support/clinical-toolkits/heart-failure-practice-solutions/left-ventricular-ejection-fraction-lvef-assessment-outpatient-setting (Accessed June 1, 2021).

[B3] Antony A. Lee T. P. (2019). Herb-induced Liver Injury with Cholestasis and Renal Injury Secondary to Short-Term Use of Kratom (Mitragyna Speciosa). Am. J. Ther. 26, e546–547. 10.1097/MJT.0000000000000802 29927773

[B4] Barbieri A. Bursi F. Mantovani F. Valenti C. Quaglia M. Berti E. (2012). Left Ventricular Hypertrophy Reclassification and Death: Application of the Recommendation of the American Society of Echocardiography/European Association of Echocardiography. Eur. Heart J. Cardiovasc. Imaging 13, 109–117. 10.1093/ejechocard/jer176 21979990

[B5] Corkery J. M. Streete P. Claridge H. Goodair C. Papanti D. Orsolini L. (2019). Characteristics of Deaths Associated with Kratom Use. J. Psychopharmacol. 33, 1102–1123. 10.1177/0269881119862530 31429622

[B6] Crawford M. H. (2017). Current Diagnosis & Treatment Cardiology. 5th ed. New York (NY): McGraw-Hill Education.

[B7] de Winter J. C. F. (2013). Using the Student's "T"-Test with Extremely Small Sample Sizes. Available from: https://eric.ed.gov/?id=EJ1015748 (Accessed June 5, 2021).

[B8] Dorman C. Wong M. Khan A. (2015). Cholestatic Hepatitis from Prolonged Kratom Use: a Case Report. Hepatology 61, 1086–1087. 10.1002/hep.27612 25418457

[B28] Eastlack S. C. Cornett E. M. Kaye A. D. (2020). Kratom-Pharmacology, Clinical Implications, and outlook: A Comprehensive Review. Pain Ther. 9 (1), 55–69. 10.1007/s40122-020-00151-x 31994019 PMC7203303

[B9] ELJack A. Beasley M. Ibrahim H. Taha M. Werns S. (2020). Kratom-associated Ventricular Fibrillation. Am. J. Ther. 1, 1. 10.1097/MJT.0000000000001134 32427615

[B10] Fernandes C. T. Iqbal U. Tighe S. P. Ahmed A. (2019). Kratom-Induced Cholestatic Liver Injury and its Conservative Management. J. Investig. Med. High Impact Case Rep. 7, 2324709619836138. 10.1177/2324709619836138 PMC6440031 30920318

[B11] Fluyau D. Revadigar N. (2017). Biochemical Benefits, Diagnosis, and Clinical Risks Evaluation of Kratom. Front. Psychiatry 8, 62. 10.3389/fpsyt.2017.00062 28484399 PMC5402527

[B12] Griffiths C. L. Gandhi N. Olin J. L. (2018). Possible Kratom-Induced Hepatomegaly: a Case Report. J. Am. Pharm. Assoc. 58, 561–563. 10.1016/j.japh.2018.05.006 30041853

[B13] Grundmann O. (2017). Patterns of Kratom Use and Health Impact in the US-Results from an Online Survey. Drug Alcohol Depend 176, 63–70. 10.1016/j.drugalcdep.2017.03.007 28521200

[B14] Hahn R. T. Thomas J. D. Khalique O. K. Cavalcante J. L. Praz F. Zoghbi W. A. (2019). Imaging Assessment of Tricuspid Regurgitation Severity. JACC Cardiovasc. Imaging 12, 469–490. 10.1016/j.jcmg.2018.07.033 30846122

[B15] Hanapi N. A. Ismail S. Mansor S. M. (2013). Inhibitory Effect of Mitragynine on Human Cytochrome P450 Enzyme Activities. Pharmacognosy Res. 5, 241–246. 10.4103/0974-8490.118806 24174816 PMC3807987

[B16] Hughes R. L. (2019). Fatal Combination of Mitragynine and Quetiapine - a Case Report with Discussion of a Potential Herb-Drug Interaction. Forensic Sci. Med. Pathol. 15, 110–113. 10.1007/s12024-018-0049-9 30498933

[B17] Leong Abdullah M. F. I. Tan K. L. Narayanan S. Yuvashnee N. Chear N. J. Y. Singh D. (2021). Is Kratom (Mitragyna Speciosa Korth.) Use Associated with ECG Abnormalities? Electrocardiogram Comparisons between Regular Kratom Users and Controls. Clin. Toxicol. (Phila) 59, 400–408. 10.1080/15563650.2020.1812627 32870119

[B18] Lu J. Wei H. Wu J. Jamil M. F. Tan M. L. Adenan M. I. (2014). Evaluation of the Cardiotoxicity of Mitragynine and its Analogues Using Human Induced Pluripotent Stem Cell-Derived Cardiomyocytes. PLoS One 9, e115648. 10.1371/journal.pone.0115648 25535742 PMC4275233

[B19] Osborne C. S. Overstreet A. N. Rockey D. C. Schreiner A. D. (2019). Drug-induced Liver Injury Caused by Kratom Use as an Alternative Pain Treatment amid an Ongoing Opioid Epidemic. J. Investig. Med. High Impact Case Rep. 7, 2324709619826167. 10.1177/2324709619826167 PMC6350132 30791718

[B20] Rosenthal L. (2020). Normal Electrocardiography (ECG) Intervals. Available from: https://emedicine.medscape.com/article/2172196-overview (Accessed July 21, 2021).

[B21] Sheikh M. Ahmed N. Gandhi H. Chen O. (2021). Report of Ventricular Fibrillation in a 44-Year-Old Man Using Kratom. BMJ Case Rep. 14, e237837. 10.1136/bcr-2020-237837 PMC7993157 33758039

[B22] Singh D. Narayanan S. Vicknasingam B. (2016). Traditional and Non-traditional Uses of Mitragynine (Kratom): A Survey of the Literature. Brain Res. Bull. 126 (Pt 1), 41–46. 10.1016/j.brainresbull.2016.05.004 27178014

[B23] Straus S. M. Kors J. A. De Bruin M. L. van der Hooft C. S. Hofman A. Heeringa J. (2006). Prolonged QTc Interval and Risk of Sudden Cardiac Death in a Population of Older Adults. J. Am. Coll. Cardiol. 47, 362–367. 10.1016/j.jacc.2005.08.067 16412861

[B24] Surawicz B. Childers R. Deal B. J. Gettes L. S. Bailey J. J. Gorgels A. (2009). AHA/ACCF/HRS Recommendations for the Standardization and Interpretation of the Electrocardiogram: Part III: Intraventricular Conduction Disturbances: a Scientific Statement from the American Heart Association Electrocardiography and Arrhythmias Committee, Council on Clinical Cardiology; the American College of Cardiology Foundation; and the Heart Rhythm Society: Endorsed by the International Society for Computerized Electrocardiology. Circulation 119, e235–e240. 10.1161/CIRCULATIONAHA.108.191095 19228822

[B25] Tay Y. L. Amanah A. Adenan M. I. Wahab H. A. Tan M. L. (2019). Mitragynine, an Euphoric Compound Inhibits hERG1a/1b Channel Current and Upregulates the Complexation of hERG1a-Hsp90 in HEK293-hERG1a/1b Cells. Sci. Rep. 9, 19757. 10.1038/s41598-019-56106-6 31874991 PMC6930223

[B26] Vandenberk B. Vandael E. Robyns T. Vandenberghe J. Garweg C. Foulon V. (2016). Which QT Correction Formulae to Use for QT Monitoring? J. Am. Heart Assoc. 5, e003264. 10.1161/JAHA.116.003264 27317349 PMC4937268

[B27] Waters M. Oxner A. Krajden S. Sultanian R. (2018). Acute Liver Injury Associated with Khat Use in a 24-Year-Old Male. Case Rep. Hepatol 2018, 2816907. 10.1155/2018/2816907 PMC6280303 30584482

