# Supplementary material for: Assessment of Cardiovascular Functioning Among Regular Kratom (Mitragyna speciosa Korth) Users: A Case Series
Source: Front Pharmacol. 2021 Aug 24;12:723567. doi: 10.3389/fphar.2021.723567 (PMC8421531; doi:10.3389/fphar.2021.723567)
Supplement: Supplementary file 1 [file Table1.docx]

**Supplementary Table 1. Detailed blood investigations of all the respondents**

| **Case** | **Complete blood count** | **Renal profile and serum electrolytes** | **Liver function test** | **Thyroid function test** | **Fasting lipid profile and fasting blood sugar** |
| --- | --- | --- | --- | --- | --- |
| Case 1 | WBC = 8.2 x 10^9^/l,  Hb= 13.6 g/dL,  platelet= 243 x 10^9^/l,  HCT= 36.2%  MCV= 84.5 fL,  MCH= 31.9 pg,  MCHC= 36.7 g/l | sodium = 139 mmol/L, potassium= 4.3 mmol/L, chloride= 101 mmol/L, urea = 3.6 mmol/L, creatinine= 88 µmol/L, calcium= 2.02 mmol/L, phosphate= 1.35 mmol/L, uric acid= 455 µmol/L | Total protein= 72 g/L, Albumin= 40 g/L, Globulin= 32 g/L,  AG ratio= 1.25,  AST= 30 U/L,  ALT= 36 U/L,  ALP= 69 U/L | T4= 100 nmol/L  TSH= 2.9 mIU/L | Total cholesterol= 3.86 mmol/L,  LDL= 1.49 mmol/L,  HDL= 1.15 mmol/L,  TG= 2.68 mmol/L (high)  FBG= 4.2 mmol/L |
| Case 2 | WBC = 8.5 x 10^9^/l,  Hb= 13.9 g/dL,  platelet= 255 x 10^9^/l,  HCT= 36.7%  MCV= 86.5 fL,  MCH= 31.7 pg,  MCHC= 35.7 g/l | sodium = 142 mmol/L, potassium= 4.0 mmol/L, chloride= 99 mmol/L,  urea = 3.2 mmol/L, creatinine= 85 µmol/L, calcium= 2.01 mmol/L, phosphate= 1.36 mmol/L, uric acid= 435 µmol/L | Total protein= 76 g/L, Albumin= 45 g/L, Globulin= 31 g/L,  AG ratio= 1.45,  AST= 36 U/L,  ALT= 33 U/L,  ALP= 59 U/L | T4= 98 nmol/L  TSH= 1.6 mIU/L | Total cholesterol= 5.81 mmol/L,  LDL= 3.66 mmol/L,  HDL= 1.39 mmol/L,  TG= 1.68 mmol/L  FBG= 4.0 mmol/L |
| Case 3 | WBC = 8.4 x 10^9^/l,  Hb= 14.2 g/dL,  platelet= 275 x 10^9^/l,  HCT= 37.7%  MCV= 85.7 fL,  MCH= 32.7 pg,  MCHC= 36.8 g/l | sodium = 140 mmol/L, potassium= 4.1 mmol/L, chloride= 100 mmol/L,  urea = 3.9 mmol/L, creatinine= 84 µmol/L, calcium= 1.99 mmol/L, phosphate= 1.37 mmol/L, uric acid= 438 µmol/L | Total protein= 71 g/L, Albumin= 40 g/L, Globulin= 31 g/L,  AG ratio= 1.29,  AST= 46 U/L,  ALT= 45 U/L,  ALP= 69 U/L | T4= 123 nmol/L  TSH= 1.4 mIU/L | Total cholesterol= 5.36 mmol/L,  LDL= 3.13 mmol/L,  HDL= 1.22 mmol/L,  TG= 2.22 mmol/L.  FBG= 3.8 mmol/L |
| Case 4 | WBC = 8.6 x 10^9^/l,  Hb= 14.1 g/dL,  platelet= 270 x 10^9^/l,  HCT= 36.8%  MCV= 86.7 fL,  MCH= 33.7 pg,  MCHC= 37.8 g/l | sodium = 142 mmol/L, potassium= 4.4 mmol/L, chloride= 98 mmol/L,  urea = 3.1 mmol/L, creatinine= 82 µmol/L, calcium= 2.02 mmol/L, phosphate= 1.34 mmol/L, uric acid= 463 µmol/L | Total protein= 74 g/L, Albumin= 41 g/L, Globulin= 33 g/L,  AG ratio= 1.24 U/L,  AST= 27 U/L,  ALT= 26 U/L,  ALP= 63 U/L | T4= 130 nmol/L  TSH= 1.2 mIU/L | Total cholesterol= 4.21 mmol/L,  LDL= 3.16 mmol/L,  HDL= 0.85 mmol/L,  TG= 5.24 mmol/L (high)  FBG= 5.0 mmol/L |
| Case 5 | WBC = 8.3 x 10^9^/l,  Hb= 13.4 g/dL,  platelet= 264 x 10^9^/l,  HCT= 36.6%  MCV= 86.9 fL,  MCH= 35.7 pg,  MCHC= 38.8 g/l | sodium = 139 mmol/L, potassium= 4.0 mmol/L, chloride= 102 mmol/L,  urea = 4.6 mmol/L, creatinine= 90 µmol/L, calcium= 2.01 mmol/L, phosphate= 1.35 mmol/L, uric acid= 443 µmol/L | Total protein= 74 g/L, Albumin= 42 g/L, Globulin= 32 g/L,  AG ratio= 1.31,  AST= 32 U/L,  ALT= 44 U/L,  ALP= 56 U/L | T4= 106 nmol/L  TSH= 2.7 mIU/L | Total cholesterol= 5.20 mmol/L (high),  LDL= 3.59 mmol/L (high), HDL= 1.25 mmol/L,  TG= 0.80 mmol/L  FBG= 4.5 mmol/L |
| Case 6 | WBC = 7.3 x 10^9^/l,  Hb= 13.6 g/dL,  platelet= 267 x 10^9^/l,  HCT= 36.2%  MCV= 85.9 fL,  MCH= 39.7 pg,  MCHC= 37.8 g/l | sodium = 141 mmol/L, potassium= 3.9 mmol/L, chloride= 96 mmol/L,  urea = 4.2 mmol/L, creatinine= 89 µmol/L, calcium= 2.03 mmol/L, phosphate= 1.33 mmol/L, uric acid= 463 µmol/L | Total protein= 70 g/L, Albumin= 40 g/L, Globulin= 30 g/L,  AG ratio= 1.31,  AST= 38 U/L,  ALT= 40 U/L,  ALP= 46 U/L | T4= 128 nmol/L  TSH= 1.3 mIU/L | Total cholesterol= 6.68 mmol/L (high),  LDL= 4.65 mmol/L (high), HDL= 1.64 mmol/L,  TG= 0.85 mmol/L  FBG= 4.2 mmol/L |
| Case 7 | WBC = 8.8 x 10^9^/l,  Hb= 13.4 g/dL,  platelet= 263 x 10^9^/l,  HCT= 37.1%  MCV= 85.5 fL,  MCH= 30.9 pg,  MCHC= 36.1 g/l | sodium = 140 mmol/L, potassium= 4.7 mmol/L, chloride= 104 mmol/L, urea = 3.2 mmol/L, creatinine= 78 µmol/L, calcium= 2.22 mmol/L, phosphate= 1.33 mmol/L, uric acid= 427 µmol/L | Total protein= 70 g/L, Albumin= 40 g/L, Globulin= 30 g/L,  AG ratio= 1.33,  AST= 25 U/L,  ALT= 23 U/L,  ALP= 50 U/L | T4= 104 nmol/L  TSH= 2.6 mIU/L | Total cholesterol= 3.96 mmol/L,  LDL= 1.44 mmol/L,  HDL= 1.08 mmol/L,  TG= 3.17 (high)  FBG= 3.2 mmol/L |
| Case 8 | WBC = 8.3 x 10^9^/l,  Hb= 13.8 g/dL,  platelet= 269 x 10^9^/l,  HCT= 36.6%  MCV= 85.7 fL,  MCH= 35.7 pg,  MCHC= 35.8 g/l | sodium = 143 mmol/L, potassium= 4.5 mmol/L, chloride= 105 mmol/L, urea = 3.9 mmol/L, creatinine= 88 µmol/L, calcium= 2.11 mmol/L, phosphate= 1.29 mmol/L, uric acid= 467 µmol/L | Total protein= 69 g/L, Albumin= 37 g/L, Globulin= 32 g/L,  AG ratio= 1.16,  AST= 30 U/L,  ALT= 38 U/L,  ALP= 49 U/L | T4= 127 nmol/L  TSH= 1.8 mIU/L | Total cholesterol= 4.95 mmol/L,  LDL= 2.80 mmol/L,  HDL= 1.85 mmol/L,  TG= 0.66 mmol/L  FBG= 3.1 mmol/L |
| Case 9 | WBC = 7.6 x 10^9^/l,  Hb= 14.0 g/dL,  platelet= 266 x 10^9^/l,  HCT= 36.8%  MCV= 85.3 fL,  MCH= 33.7 pg,  MCHC= 35.4 g/l | sodium = 140 mmol/L, potassium= 4.2 mmol/L, chloride= 103 mmol/L, urea = 4.9 mmol/L, creatinine= 86 µmol/L, calcium= 2.04 mmol/L, phosphate= 1.33 mmol/L, uric acid= 487 µmol/L | Total protein= 74 g/L, Albumin= 40 g/L, Globulin= 34 g/L,  AG ratio= 1.18,  AST= 32 U/L,  ALT= 36 U/L,  ALP= 59 U/L | T4= 116 nmol/L  TSH= 2.0 mIU/L | Total cholesterol= 4.23 mmol/L,  LDL= 2.56 mmol/L,  HDL= 1.24 mmol/L,  TG= 1.05 mmol/L.  FBG= 3.6 mmol/L |

WBC= white blood cell, Hb= hemoglobin, HCT= hematocrit, MCV= mean corpuscular volume, MCH= mean corpuscular hemoglobin, MCHC= mean corpuscular hemoglobin concentration, AG ratio= albumin globulin ratio, AST= aspartate transaminase, ALT= alanine transaminase, ALP= alkaline phosphatase, LDL= low density lipoprotein, HDL= high density lipoprotein, TG= triglyceride, FBS= fasting blood glucose, T4= total thyroxine, TSH= thyroid stimulating hormone
