# Supplementary material for: Assessment of Cardiovascular Functioning Among Regular Kratom (Mitragyna speciosa Korth) Users: A Case Series
Source: Front Pharmacol. 2021 Aug 24;12:723567. doi: 10.3389/fphar.2021.723567 (PMC8421531; doi:10.3389/fphar.2021.723567)
Supplement: Supplementary file 2 [file Presentation1.pdf]

Analysis of Mitragynine in Blood Samples using Liquid Chromatography-Tandem Mass Spectrometry (LC-MS/MS)

Table of Contents

INTRODUCTION **2**

MATERIALS AND METHODS **3**

Chemicals and Standards 3

Materials 3

Instrumentation 3

Instrument Parameters 4

Analyte MS/MS Fragmentation Parameters 5

Mobile phase A: 0.1% Formic Acid in 10 mM Ammonium Formate 6

Mobile phase B: 0.1% Formic Acid in Acetonitrile 6

Sample Preparation and Solid-Phase Extraction 6

RESULTS **8**

REFERENCES **13**

APPENDIX **14**

Appendix 1: MRM Mitragynine Reference Standard 14

Appendix 2: MRM Sample 107553 18

Appendix 3: MRM Sample 107558 22

Appendix 4: MRM Sample 107559 26

Appendix 5: MRM Sample 107561 30

Appendix 6: MRM Sample 107563 34

Appendix 7: MRM Sample 107564 38

Appendix 8: MRM Sample 107565 42

Appendix 9: MRM Sample 107566 46

Appendix 10: MRM Sample 107567 50

INTRODUCTION

A method using solid-phase extraction (SPE) and liquid chromatography-tandem mass spectrometry was performed to detect and quantify for mitragynine in 15 human serum samples. These samples were submitted to USAINS Biomics Laboratory Testing Services Sdn Bhd (BIOMICS) by the Centre for Drug Research, Universiti Sains Malaysia (USM). The sample extraction procedure was modified from a published and validated method by Lee, M.J. (2016) and Lee *et al*. (2018). Mitragynine was identified with multiple reaction monitoring (MRM) in the positive electrospray ionization (ESI) mode using nalorphine as the internal standard (IS).

A batch of 9 human serum samples were submitted for analysis and quantification of mitragynine. These samples were subjected to solid-phase extraction (SPE) and liquid chromatography-tandem mass spectrometry (LC-MS/MS) analysis modified from a method validated by Lee, M.J. (2018).

No.	Laboratory Code	Sample ID
1	107553	10039288
2	107554	10039286
3	107559	10039287
4	107561	20038840
5	107563	10040642
6	107564	10040935
7	107565	10040906
8	107566	10040890
9	107567	10040891

Table 1: Sample identification and coding for the 9 human serum samples received for mitragynine analysis.

MATERIALS AND METHODS

Chemicals and Standards

Chemicals	Company / Source
Mitragynine	Chemtron Biotechnology Sdn Bhd, Malaysia.
Nalorphine	Cerilliant Corporation,<br>Round Rock, Texas
Methanol (HPLC grade)	Fischer Scientific, UK.
Acetonitrile (ACN) HPLC grade	Merck KGaA, Darmstadt, Germany
Ammonium formate	Fluka, Switzerland
Formic aicd, OPTIMA LC/MS	Fischer Scientific, Fair Lawn, NJ07410
Ammonium hydroxide	J.T. Baker®, USA
Hydrochloric acid	Fischer Chemicals, USA
Formic acid<br>Puriss p.a. eluent additive for LC-MS ~ 98% (T)	Fluka Analytical, Germany, Sigma-Aldrich Corporation
Methanol (HPLC grade)	Fisher Scientific UK

Materials

Materials Description	Company / Source
Bond-Elut Certify SPE (100 mg, 1 mL)	Agilent Technologies Inc., USA
SB-C18 Guard Cartridge<br>Dimension: 4.6 mm x 12.5 mm, 5 µm	Agilent Technologies Inc., USA
ZORBAX Eclipse XDB-C18 HPLC Column<br>Dimension: 4.6 mmm x 150 mm, 5 µm	Agilent Technologies Inc., USA

Instrumentation

Instrument Description	Company / Source
Agilent Technologies 1290 Infinity II High-Performance Liquid Chromatograph (HPLC) with quaternary pump	Agilent Technologies Inc., USA
Liquid Chromatography-Mass Spectrometry (LC-MS) System<br>Model: 6470 Triple Quad LC/MS with 1290 Infinity II HPLC using Agilent Infinity II 1290 quaternary pump	Agilent Technologies Inc., USA

Instrument Parameters

The following table showed the instrument parameters used for the Agilent HPLC and LC-MS System for the analysis of mitragynine.

Instrument Parameter	Parameter Description
Sample injection volume:	5 µL
Column compartment temperature:	35^0C
Mobile phases:	**A**: 0.1% formic acid in 10 mM ammonium formate<br>**B**: 0.1% formic acid in acetonitrile
Solvent gradient programme:	(see gradient table below)<br>Post-run time: 2 minutes
MS/MS conditions:	Gas temperature: 300^0C<br>Gas flow: 5 L/min<br>Nebulizer pressure: 45 psi<br>Sheath gas temperature: 250^0C<br>Sheath gas flow: 11 L/min<br>Capillary voltage: 3500 V<br>Nozzle voltage: 500 V<br>MS1 temperature: 100^0C<br>MS2 temperature: 100^0C

Solvent gradient programme:

Time (minute)	Mobile Phase		Flow rate (mL/min)
	% A	% B	
0	90	10	1.0
9	0	100	1.0
12	0	100	1.0
12.2	90	10	1.5
20	90	10	1.5

Post-run time: 2 minutes

Table 2: The Agilent HPLC and 6470 Triple Quad LC-MS instrument parameters for the analysis of mitragynine in human serum sample.

Analyte MS/MS Fragmentation Parameters

Analyte	Precursor ion	Product ion	Collision energy (CE)	Fragmentor voltage (V)
Mitragynine 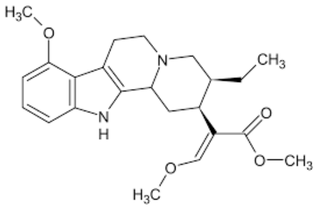	399.2	174.1	21	176
		238.1	29	176
		226.1	29	176
		159.1	50	176
Nalorphine 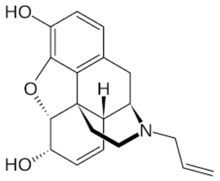	312.2	152.1	74	62
		77.1	110	62

The following figure is a proposed fragmentation and structural rearrangements pathway of mitragynine.

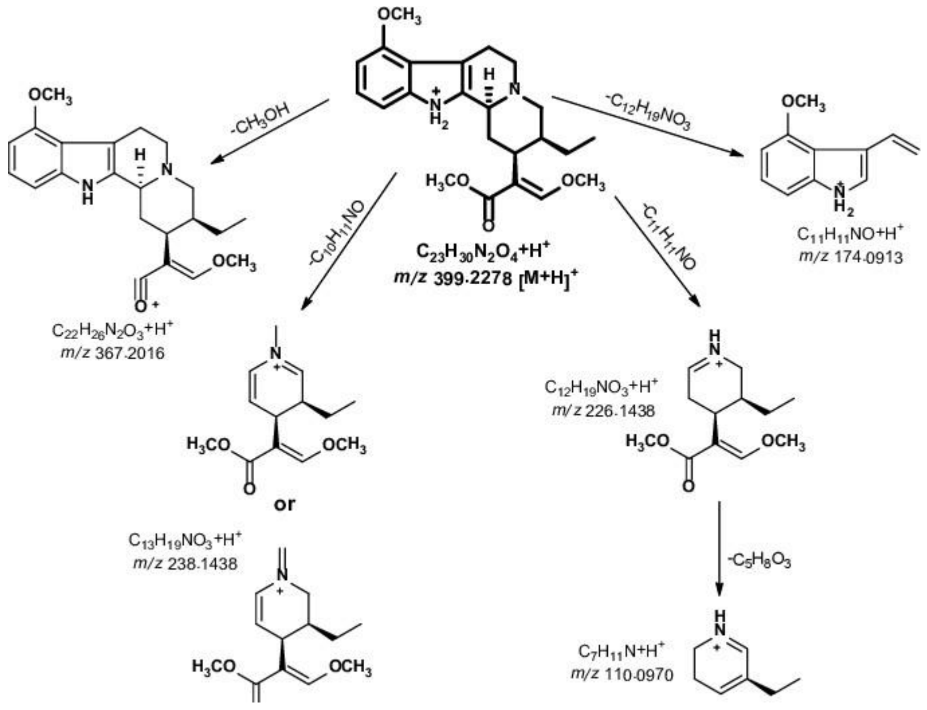

Figure 1: Proposed fragmentation and structural rearrangements pathway of mitragynine (Avula et al., 2015).

Mobile Phase A: **0.1% Formic Acid in 10 mM Ammonium Formate**

Weigh 0.63 g ammonium formate into a 1 L volumetric flask and dissolve in approximately 500 mL of deionised water. Then, add 1 mL of formic acid into the solution and make up volume to 1 L with deionised water.

Mobile Phase B: **0.1% Formic Acid in Acetonitrile**

Dispense 1 mL of formic acid into a 1 L volumetric flask and make up volume to 1 L with acetonitrile.

Sample Preparation and Solid-Phase Extraction

In this experiment, nalorphine was used as the internal standard to correct for extraction efficiency and helped to improve the method accuracy and precision. The mitragynine 1 mg/mL stock solution was prepared in duplicates (stock solution A and stock solution B). One stock was used for calibration standards preparation and another stock used for quality control purposes.

The 1 mg/mL mitragynine stock solution A was diluted to 1 µg/mL, 10 µg/mL, and 100 µg/mL working solutions with methanol to prepare the series of calibrators.

Calibrator	Calibrator Concentration (ng/mL)	Concentration of Working Solution A (µg/mL)	Volume of Working Solution Required (µL)	Volume of Blank Serum Sample (µL)
C0	0	-	-	1,000
C1	10	1	10	990
C2	100	10	10	990
C3	250	10	25	975
C4	500	100	5	995
C5	750	100	7.5	993
C6	1,000	100	10	990

Table 3: Table summarizing the preparation of the series of calibrators in blank serum matrix.

The 1 mg/mL mitragynine stock solution B was diluted to 10 µg/mL and 100 µg/mL working solutions with methanol to be used for spiking into blank serum sample.

QC Sample	Calibrator Concentration (ng/mL)	Concentration of Working Solution B (µg/mL)	Volume of Working Solution Required (µL)	Volume of Blank Serum Sample (µL)
QC-L	55	10	5.5	995
QC-M	375	10	37.5	963
QC-H	875	100	8.75	992

Table 4: Table summarizing the preparation of the 3 levels of QC in blank serum matrix.

All the coded serum samples were mixed well and centrifuged at 3,000 rpm at room temperature for 5 minutes. The same was applied to the series of calibrators and QC samples.

A 100 µL of each calibrator and QC sample was dispensed into the respective microcentrifuge tube and added with 2.5 µL of 10 µg/mL nalorphine (internal standard). They were mixed thoroughly ready to be loaded onto the solid-phase extraction (SPE) cartridges.

A 100 µL of the centrifuged serum samples were aliquoted into the respective microcentrifuge tubes and diluted with 900 µL of blank serum. The diluted serum samples were mixed thoroughly and 100 µL of each diluted sample was transferred to a new microcentrifuge. Each sample was then added with 2.5 µL of 10 µg/mL nalorphine and vortexed to mix well ready to be loaded onto the SPE cartridges.

The Bond-Elut Certify II Solid-Phase Extraction cartridges were conditioned sequentially with 1 mL of methanol and distilled water. Then, 100µL of the respective calibrators, QC samples, and subject test samples were loaded onto the assigned SPE cartridges. The SPE cartridges were then washed with 1 mL of distilled water followed by 10 mM hydrochloric acid, and finally methanol. The cartridges were then vacuum-dried before eluting with freshly prepared 2% (v/v) ammonium hydroxide in methanol (elution solvent). The eluent was dried at 60^0C under a gentle stream of nitrogen. The dried sample was reconstituted with 100 µL of the starting mobile phase (mobile phase A : mobile phase B, 90:10, v/v) and filtered prior to analysis with the Agilent Technologies 6470 Triple Quad LC/MS System coupled with the Agilent Technologies Infinity II 1290 High Performance Liquid Chromatography System (LC-MS/MS).

RESULTS

The following table summarized the concentration (µg/mL) of mitragynine detected in the 9 human plasma samples using the extraction method mentioned in the previous section. Each sample was subjected to a 100 times dilution. Therefore, the final concentrations reported in table 5 were after taking the dilution factor into consideration.

No.	LaboratoryCode	Sample ID	Mitragynine Concentrationin serum (µg/mL)
1	107553	10039288	11.3
2	107554	10039286	9.6
3	107559	10039287	20.4
4	107561	20038840	22.4
5	107563	10040642	3.6
6	107564	10040935	8.5
7	107565	10040906	6.8
8	107566	10040890	2.5
9	107567	10040891	8.0

Table 5: Summary of analysis for the detection of mitragynine in 9 human serum samples. *ND – Not detected.

A typical MRM chromatogram for mitragynine reference standard was depicted in figure 2.

A typical MRM chromatogram for mitragynine in human serum sample was shown in figure 3. Mitragynine isomers (i.e. speciogynine, mitracilliatine, speciocilliatine, paynantheine, and isopaynantheine) might contribute to the chromatographic peaks after the mitragynine chromatographic peak. However, the data was non conclusive since the reference standards for the isomers were not available.

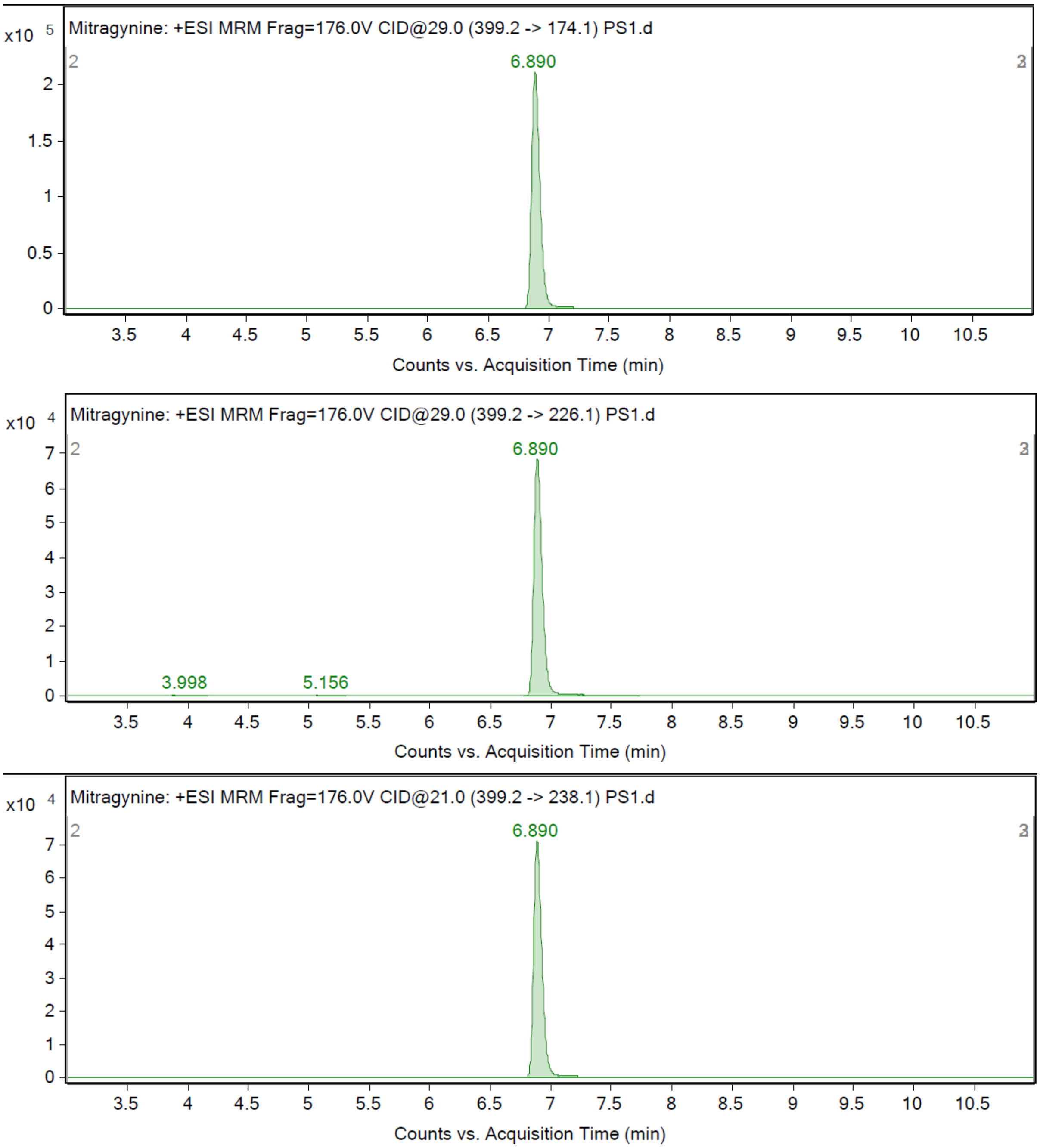

Figure 2: Typical MRM chromatogram of mitragynine reference standard using the ZORBAX Eclipse XDB-C18 HPLC column (4.6 mm x 150 mm; 5 µm).

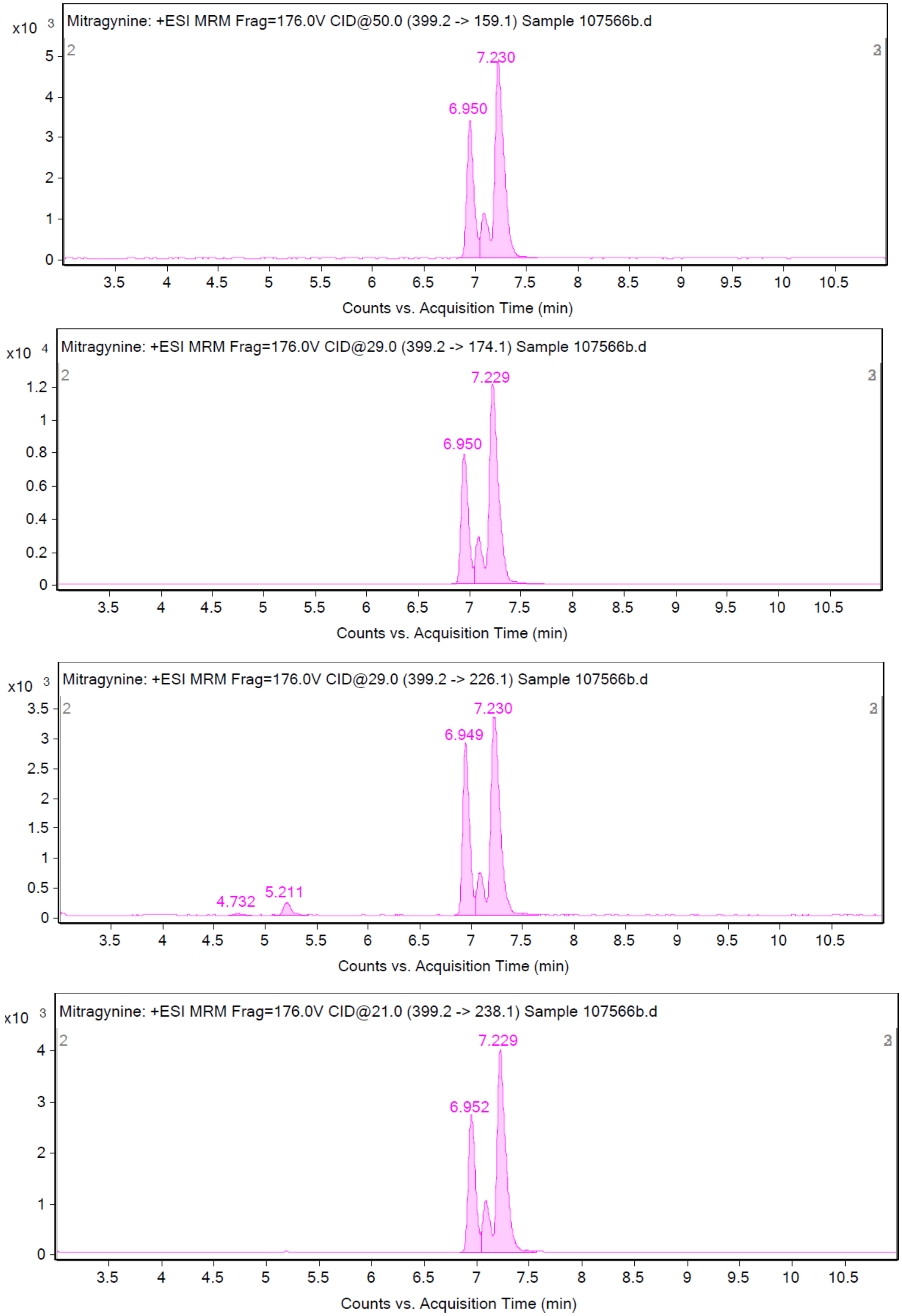

Figure 3: Typical MRM chromatogram of mitragynine in human serum sample using the ZORBAX Eclipse XDB-C18 HPLC column (4.6 mm x 150 mm; 5 µm).

The calibration curve for calculating the 15 human serum samples were as shown in figure 4 below.

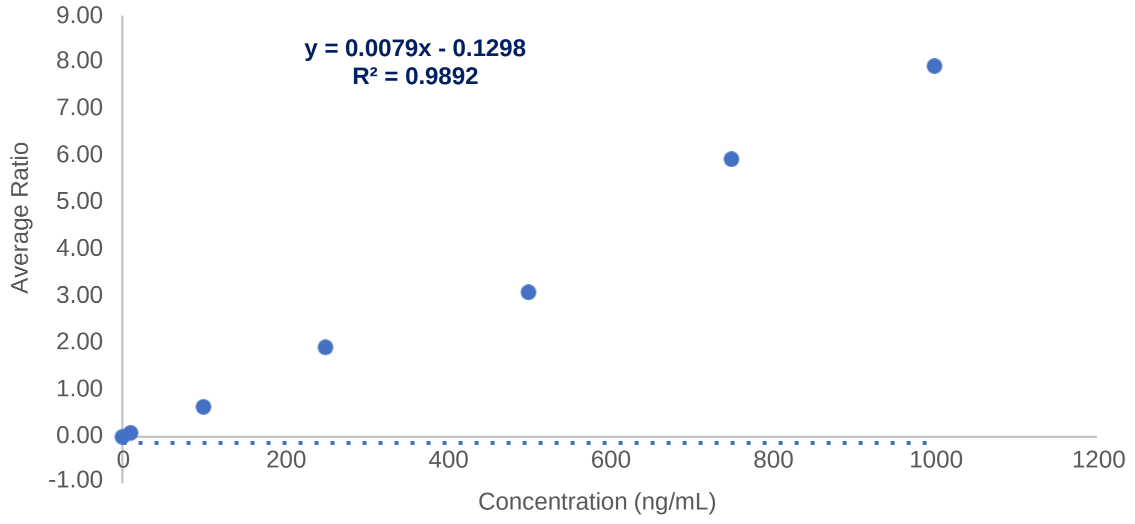

Figure 4: Typical MRM chromatogram of mitragynine reference standard using the ZORBAX Eclipse XDB-C18 HPLC column (4.6 mm x 150 mm; 5 µm).

The raw data for the calibration curve, QC levels, and 15 human serum samples were as shown in the table below.

Calibrator	Concentration (ng/mL)	AUC		Ratio	Average Ratio
		Mitragynine	Nalorphine (IS)		
C0	0	0	599,030	0.00	0.00
		0	515,985	0.00	
C1	10	58,356	590,624	0.10	0.08
		37,601	569,424	0.07	
C2	100	448,965	639,946	0.70	0.64
		421,499	727,067	0.58	
C3	250	1,395,946	724,915	1.93	1.91
		1,121,241	590,050	1.90	
C4	500	2,223,959	735,630	3.02	3.09
		2,276,643	722,208	3.15	
C5	750	2,484,201	495,904	5.01	5.94
		3,911,292	569,613	6.87	
C6	1,000	6,200,765	706,166	8.78	7.92
		4,496,430	636,970	7.06	

Table 6: Chromatographic area under the curve (AUC) data for the mitragynine calibration curve shown in figure 4.

Sample ID	Conc. (ng/mL)	AUC		Ratio	Calculated Conc. (ng/mL)	Final Conc. (ng/mL)	% Recovery
		Mitragynine	Nalorphine (IS)				
QC-L	0	199,606	650,844	0.31	55.25	56.90	103.5
		173,033	520,096	0.33	58.54		
QC-M	10	1,483,402	575,035	2.58	342.97	330.26	88.1
		1,557,071	654,538	2.38	317.56		
QC-H	100	3,387,103	604,964	5.60	725.15	778.85	89.0
		3,452,392	535,468	6.45	832.56		

Table 6: Chromatographic area under the curve (AUC) data for the mitragynine calibration curve shown in figure 4.

Sample ID	AUC		Ratio	Calculated Conc. (ng/mL)	Final Conc. (ng/mL)
	Mitragynine	Nalorphine (IS)			
107553	462,534	606,406	0.76	112.98	11,298.06
107554	397,323	629,384	0.63	96.34	9,634.03
107555	-	776,545	-	-	-
107556	261,734	561,029	0.47	75.48	7,548.42
107557	279,886	522,271	0.54	84.27	8,426.61
107558	466,885	610.617	0.76	113.22	11,321.67
107559	768,454	517,878	1.48	204.26	20,425.97
107560	1,884,687	538,746	3.50	459.25	45,925.13
107561	908,487	552,172	1.65	224.70	22,469.58
107562	640,183	575,793	1.11	157.17	15,716.81
107563	82,000	522,649	0.16	36.29	3,629.03
107564	358,446	666,446	0.54	84.51	8,451.23
107565	242,210	596,805	0.41	67.80	6,780.31
107566	39,247	575,859	0.07	25.06	2,505.74
107567	372,319	736,799	0.51	80.39	8,039.49

Table 7: Chromatographic area under the curve (AUC) data for mitragynine in the 15 human serum samples.

REFERENCES

Avula, B., Wang, Y.H., Sagi, S., and Wang, M. (2015). Identification and characterization of indole and oxindole alkaloids from leaves of Mitragyna speciosa Korth using liquid chromatography-Accurate QToF Mass Spectrometry. Journal of AOAC International. 98(1): 13-21.

Lee, M.J. (2016). Development of an immunoassay for mitragynine. Ph.D. Thesis. Universiti Sains Malaysia.

Lee, M.J., Ramanathan, S., Mansor, S.M., Yeong, K.Y., and Tan, S.C. (2018). Method validation in quantitative analysis of phase I and phase II metabolites of mitragynine in human urine using liquid chromatography-tandem mass spectrometry. Analytical Biochemistry. 543: 146-161.

Philipp, A.A., Wissenbach, D.K., Weber, A.A., Zapp, J., and Maurer, H.H. (2011). Metabolism studies of the *Kratom* alkaloids mitraciliatine and isopaynantheine, diastereoisomers of the main alkaloids mitragynine and paynantheine, in rat and human urine using liquid-chromatography-linear ion trap-mass spectrometry. Journal of Chromatography B. 879(15-16): 1049-1055.

APPENDIX

Appendix 1: MRM Mitragynine Reference Standard

Qualitative Analysis Report

**Data File**	PS1.d	**Sample Name**	PS
**Sample Type**	Calibration	**Position**	P2-B9
**Instrument Name**	Instrument 1	**User Name**	
**Acq Method**	Mitragynine_LMJ_Flexi_2.m	**Acquired Time**	31/10/2020 3:00:36 PM (UTC+08:00)
**IRM Calibration Status**	Not Applicable	**DA Method**	APCI Chemstation.m
**Comment**			
**Sample Group**		**Info.**	
**Stream Name**	LC 1	**Acquisition Time (Local)**	31/10/2020 3:00:36 PM (UTC+08:00)
**Acquisition SW Version**	6400 Series Triple Quadrupole B.08.02 (B8260.0)		

Chromatograms

**Fragmentor Voltage**	17 6	**Collision Energy**	5 0	**Ionization Mode**	ES I

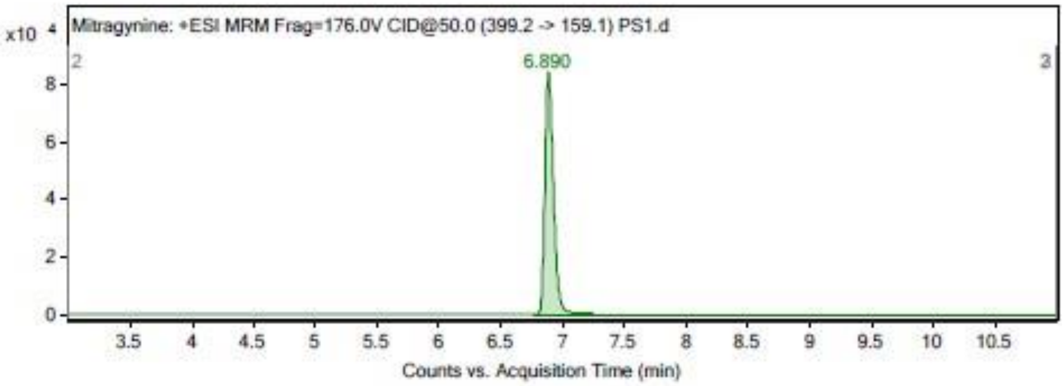

Integration Peak List

Peak	Start	RT	End	Height	Area	Area %
1	6.77	6.89	7.634	83621	414496	100

**Fragmentor Voltage**	17 6	**Collision Energy**	2 9	**Ionization Mode**	ES I

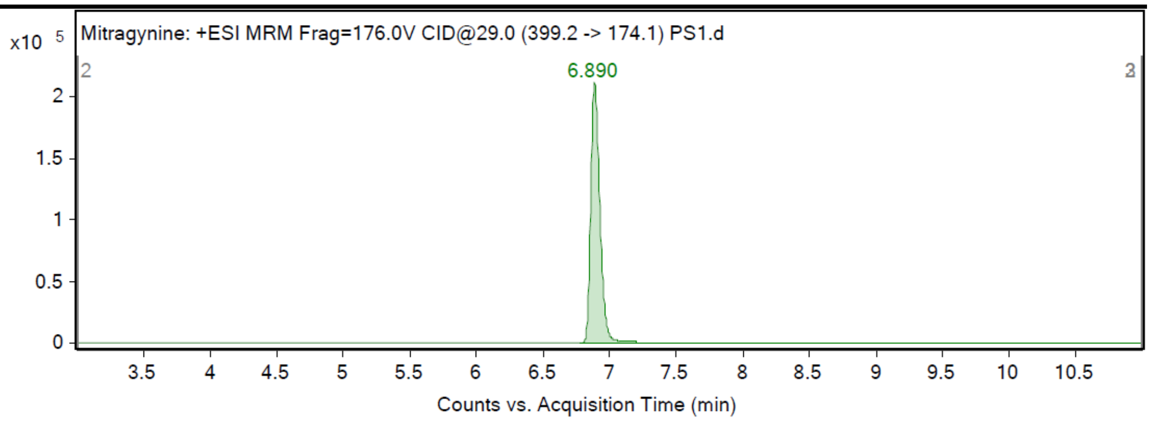

Integration Peak List

Peak	Start	RT	End	Height	Area	Area %
1	6.771	6.89	7.809	209357	1032373	100

**Fragmentor Voltage**	176	**Collision Energy**	29	**Ionization Mode**	ESI

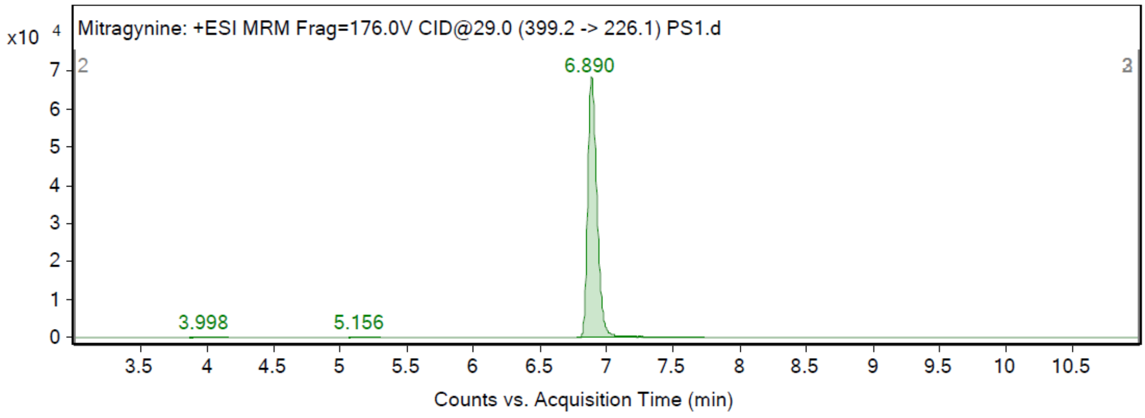

Integration Peak List

Peak	Start	RT	End	Height	Area	Area %
1	3.874	3.998	4.153	33	208	0.06
2	5.061	5.156	5.297	133	565	0.17
3	6.771	6.89	7.729	68075	338147	100

**Fragmentor Voltage**	176	**Collision Energy**	21	**Ionization Mode**	ESI

Qualitative Analysis Report

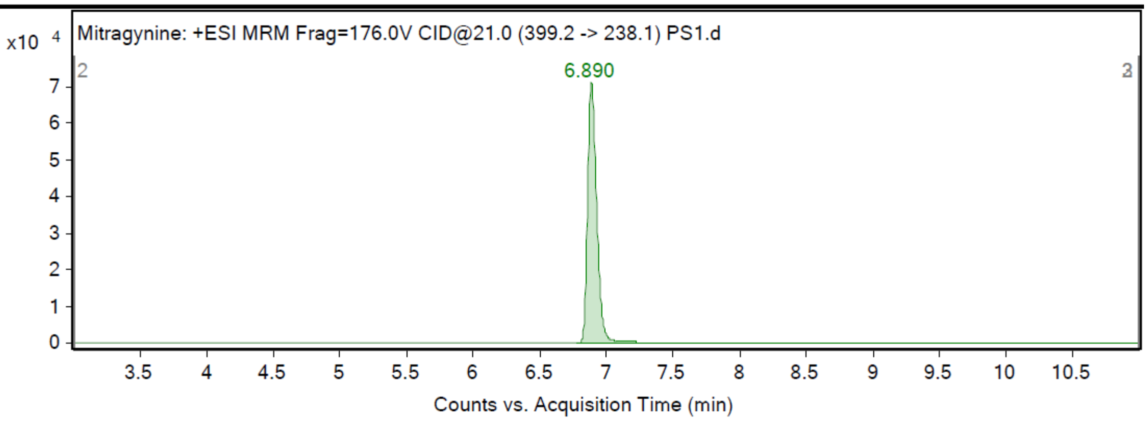

Integration Peak List

Peak	Start	RT	End	Height	Area	Area %
1	6.771	6.89	7.698	70571	345910	100

Fragmentor Voltage	62	Collision Energy	110	Ionization Mode	ESI

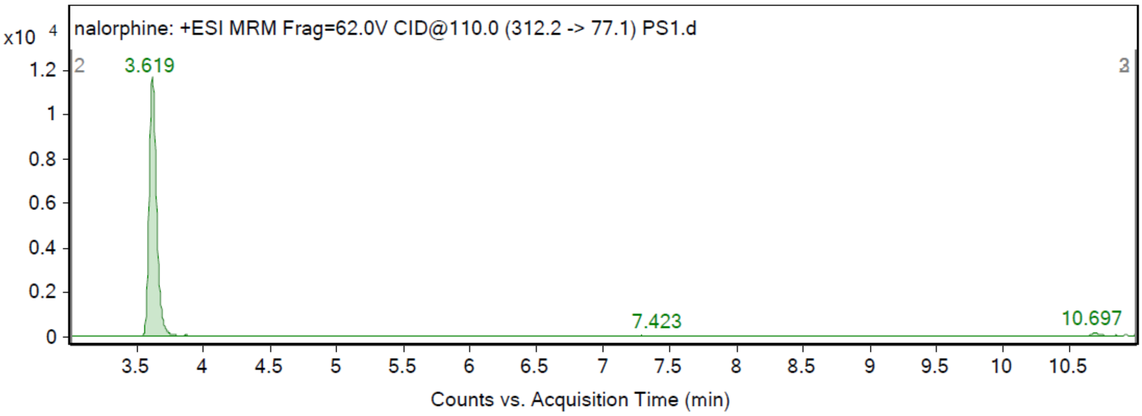

Integration Peak List

Peak	Start	RT	End	Height	Area	Area %
1	3.5	3.619	4.172	11514	46983	100
2	7.28	7.423	7.621	25	136	0.29
3	10.577	10.697	10.864	110	557	1.18

Fragmentor Voltage	62	Collision Energy	74	Ionization Mode	ESI

Qualitative Analysis Report

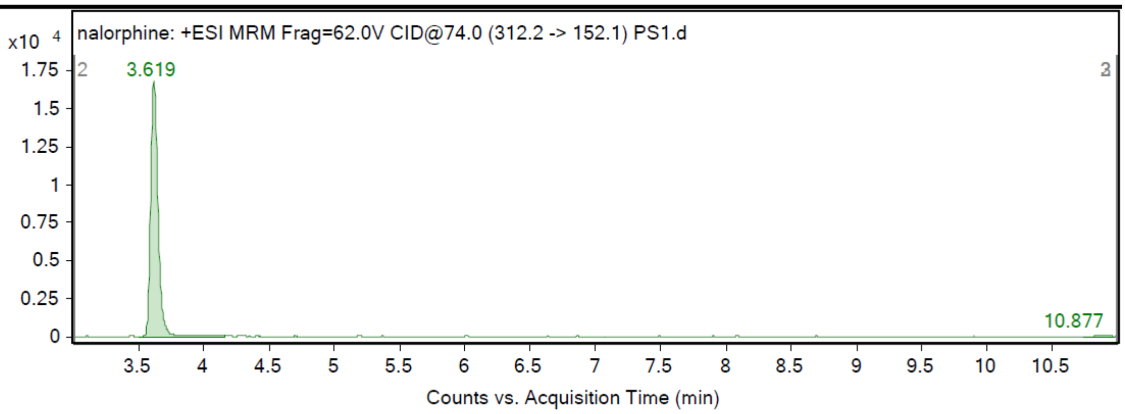

Integration Peak List

Peak	Start	RT	End	Height	Area	Area %
1	3.5	3.619	4.156	16599	67409	100
2	10.733	10.877	10.975	55	296	0.44

--- End Of Report ---

Appendix 2: MRM Sample 107553

Qualitative Analysis Report

**Data File**	Sample 107553b.d	**Sample Name**	Sample 107553b
**Sample Type**	Sample	**Position**	P2-C2
**Instrument Name**	Instrument 1	**User Name**	
**Acq Method**	Mitragynine_LMJ_Flexi_2.m	**Acquired Time**	31/10/2020 3:56:17 AM (UTC+08:00)
**IRM Calibration Status**	Not Applicable	**DA Method**	APCI Chemstation.m
**Comment**			
**Sample Group**		**Info.**	
**Stream Name**	LC 1	**Acquisition Time (Local)**	31/10/2020 3:56:17 AM (UTC+08:00)
**Acquisition SW Version**	6400 Series Triple Quadrupole B.08.02 (B8260.0)		

Chromatograms

**Fragmentor Voltage**	176	**Collision Energy**	50	**Ionization Mode**	ESI

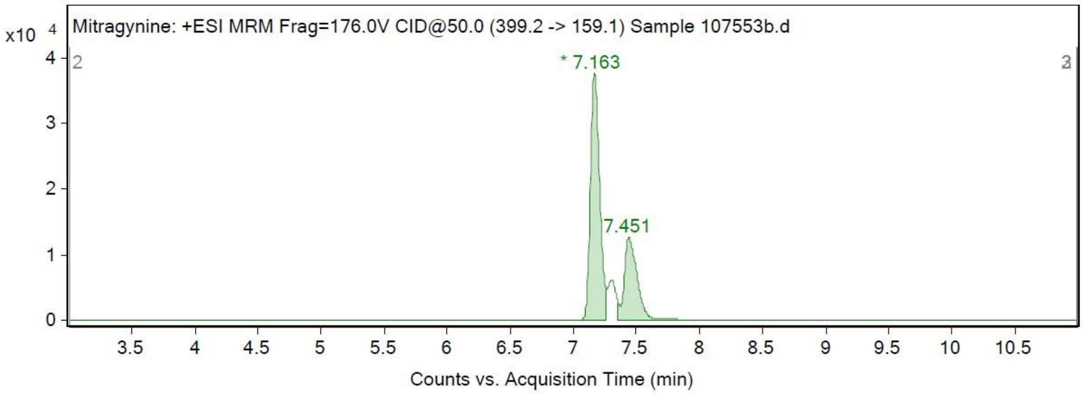

Integration Peak List

Peak	Start	RT	End	Height	Area	Area %
1	7.044	7.163	7.262	37677	192098	100
2	7.358	7.451	7.984	12599	87695	45.65

**Fragmentor Voltage**	176	**Collision Energy**	29	**Ionization Mode**	ESI

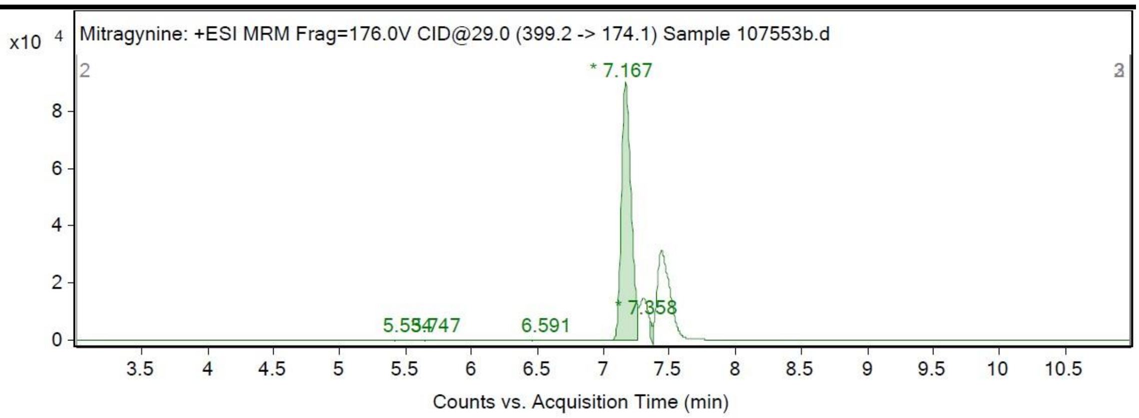

Integration Peak List

Peak	Start	RT	End	Height	Area	Area %
1	5.415	5.534	5.643	85	394	0.09
2	5.643	5.747	6.044	40	360	0.08
3	6.448	6.591	6.791	30	181	0.04
4	7.056	7.167	7.262	90397	462534	100
5	7.358	7.358	7.378	5033	6401	1.38

Fragmentor Voltage	176	Collision Energy	29	Ionization Mode	ESI

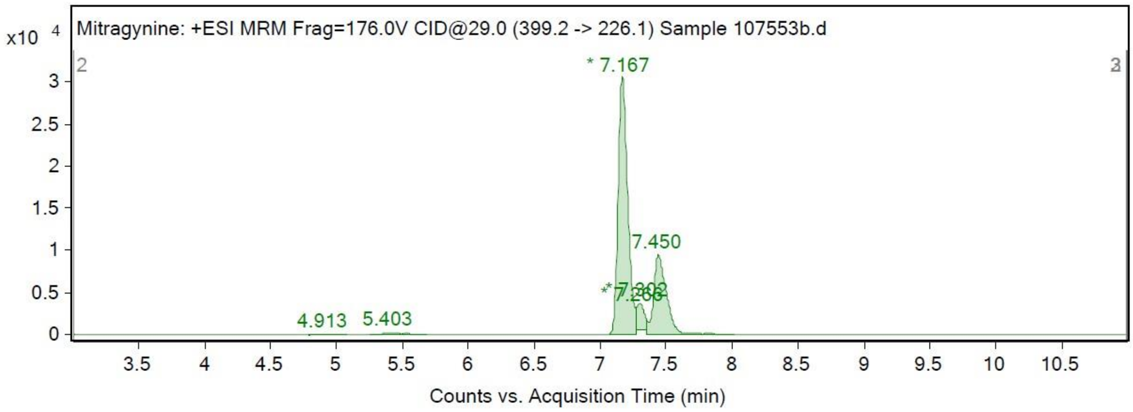

Integration Peak List

Peak	Start	RT	End	Height	Area	Area %
1	4.794	4.913	5.075	34	129	0.08
2	5.26	5.403	5.679	186	1381	0.9
3	7.072	7.167	7.266	30641	153987	100
4	7.266	7.266	7.266	3170	0	0
5	7.274	7.302	7.354	3139	11678	7.58
6	7.355	7.45	8.015	9445	62890	40.84

Fragmentor Voltage	176	Collision Energy	21	Ionization Mode	ESI

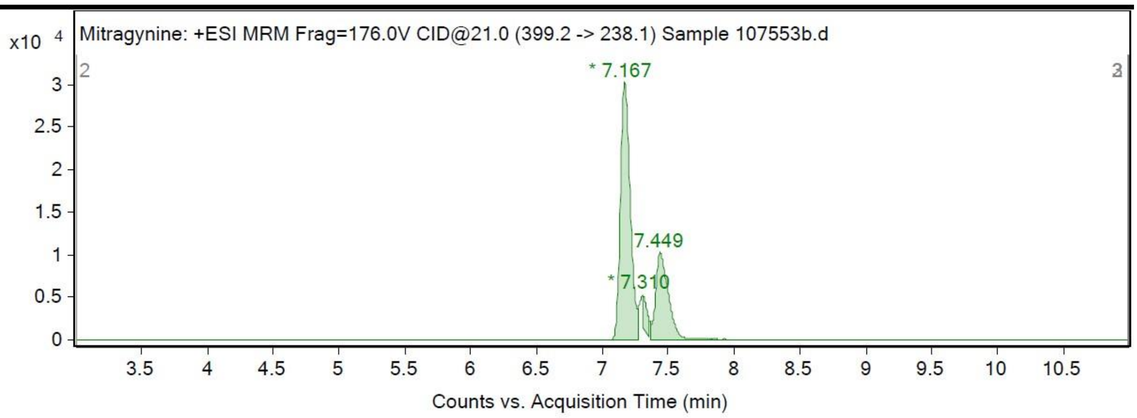

Integration Peak List

Peak	Start	RT	End	Height	Area	Area %
1	7.068	7.167	7.266	30347	155950	100
2	7.31	7.31	7.358	3777	8029	5.15
3	7.359	7.449	8.047	10159	68718	44.06

Fragmentor Voltage	62	Collision Energy	110	Ionization Mode	ESI

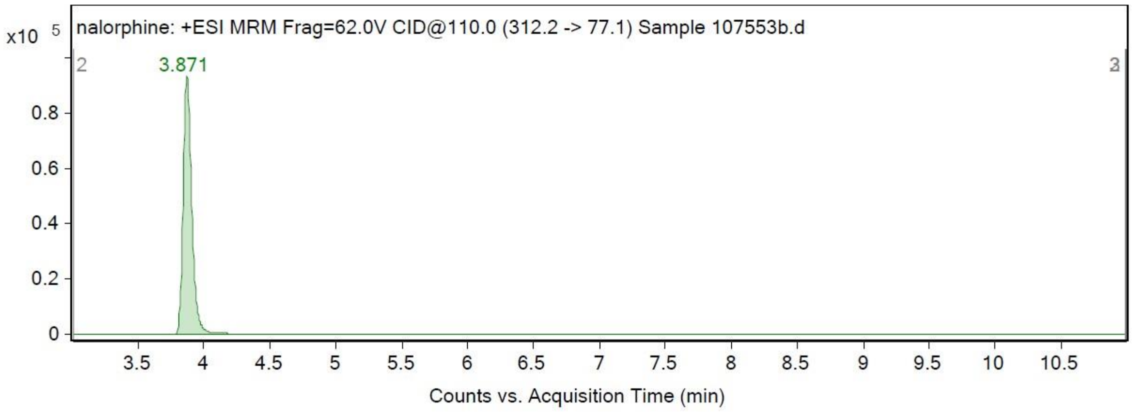

Integration Peak List

Peak	Start	RT	End	Height	Area	Area %
1	3.751	3.871	4.585	92023	426995	100

Fragmentor Voltage	62	Collision Energy	74	Ionization Mode	ESI

Qualitative Analysis Report

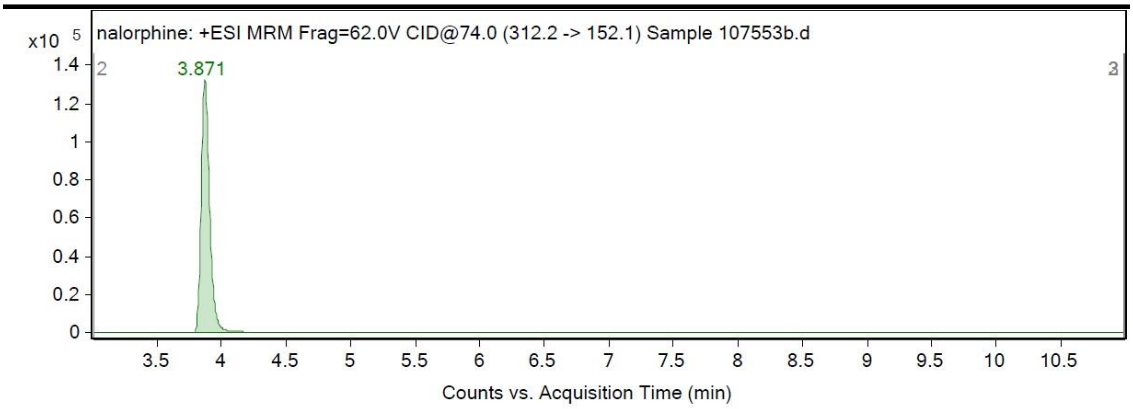

Integration Peak List

Peak	Start	RT	End	Height	Area	Area %
1	3.752	3.871	4.617	130682	606406	100

--- End Of Report ---

Appendix 3: MRM Sample 107554

Qualitative Analysis Report

**Data File**	Sample 107554b.d	**Sample Name**	Sample 107554b
**Sample Type**	Sample	**Position**	P2-C4
**Instrument Name**	Instrument 1	**User Name**	
**Acq Method**	Mitragynine_LMJ_Flexi_2.m	**Acquired Time**	31/10/2020 4:40:34 AM (UTC+08:00)
**IRM Calibration Status**	Not Applicable	**DA Method**	APCI Chemstation.m
**Comment**			
**Sample Group**		**Info.**	
**Stream Name**	LC 1	**Acquisition Time (Local)**	31/10/2020 4:40:34 AM (UTC+08:00)
**Acquisition SW Version**	6400 Series Triple Quadrupole B.08.02 (B8260.0)		

Chromatograms

**Fragmentor Voltage**	**Collision Energy**	**Ionization Mode**
176	50	ESI

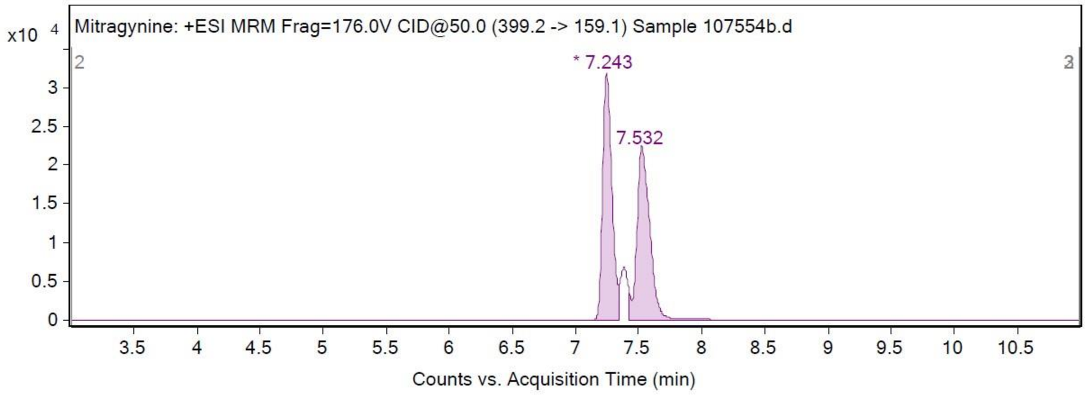

Integration Peak List

Peak	Start	RT	End	Height	Area	Area %
1	7.127	7.243	7.338	31889	162513	100
2	7.428	7.532	8.254	20853	157531	96.93

**Fragmentor Voltage**	**Collision Energy**	**Ionization Mode**
176	29	ESI

Qualitative Analysis Report

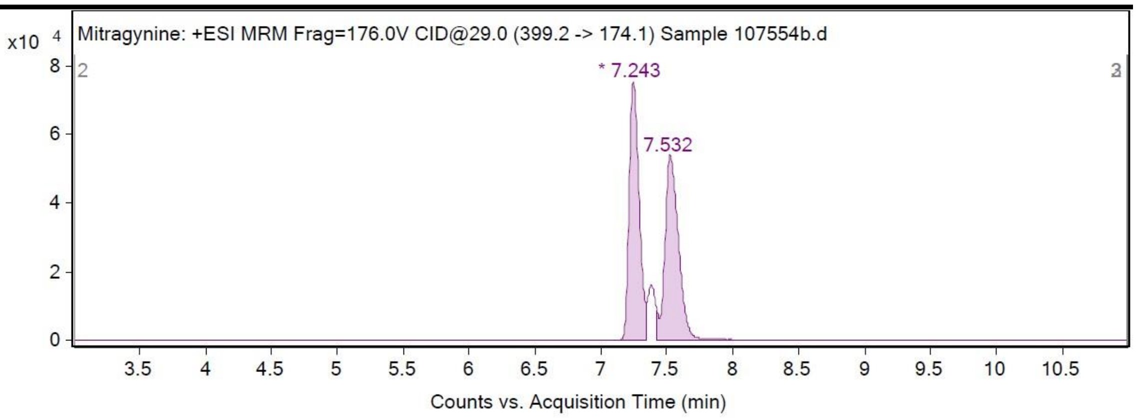

Integration Peak List

Peak	Start	RT	End	Height	Area	Area %
1	7.048	7.243	7.338	75421	397323	100
2	7.427	7.532	8.572	51058	387023	97.41

Fragmentor Voltage	17 6	Collision Energy	2 9	Ionization Mode	ES I

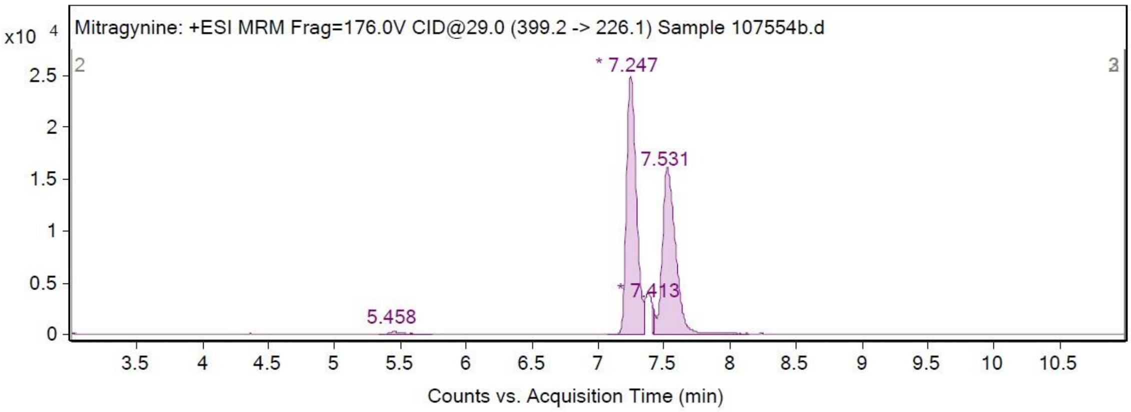

Integration Peak List

Peak	Start	RT	End	Height	Area	Area %
1	5.339	5.458	5.742	200	1168	0.89
2	7.076	7.247	7.35	24925	131039	100
3	7.413	7.413	7.421	2758	1090	0.83
4	7.424	7.531	8.143	15035	111645	85.2

Fragmentor Voltage	17 6	Collision Energy	2 1	Ionization Mode	ES I

Qualitative Analysis Report

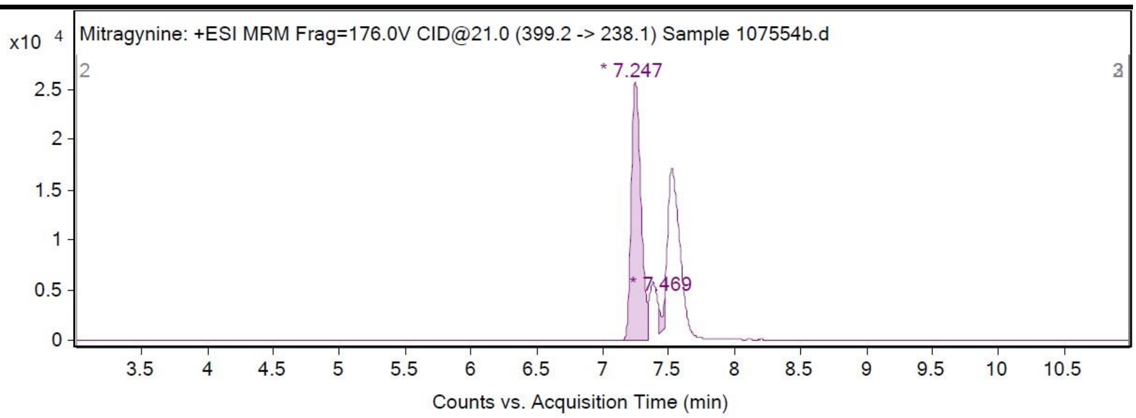

Integration Peak List

Peak	Start	RT	End	Height	Area	Area %
1	7.119	7.247	7.342	25820	133733	100
2	7.429	7.469	7.469	2954	4319	3.23

Fragmentor Voltage	6<br>2	Collision Energy	11<br>0	Ionization Mode	ES<br>I

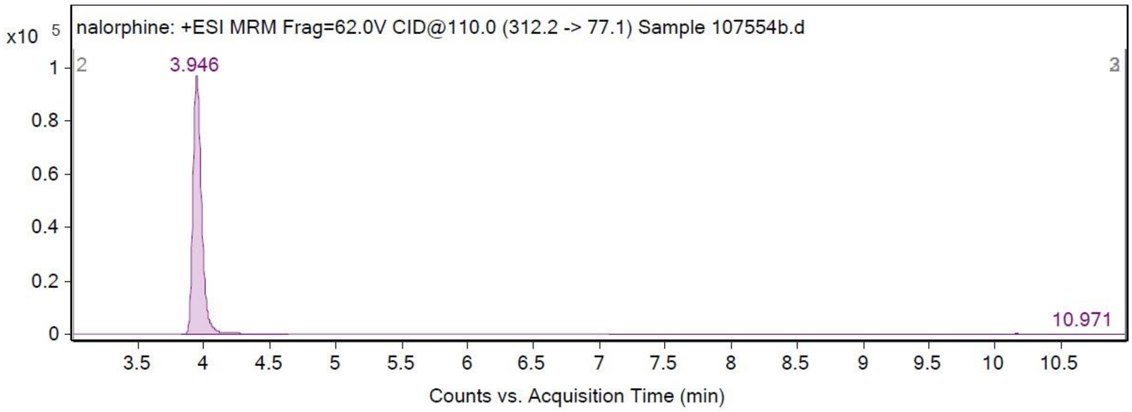

Integration Peak List

Peak	Start	RT	End	Height	Area	Area %
1	3.827	3.946	4.633	93563	448460	100
2	10.161	10.971	10.992	33	727	0.16

Fragmentor Voltage	6<br>2	Collision Energy	7<br>4	Ionization Mode	ES<br>I

Qualitative Analysis Report

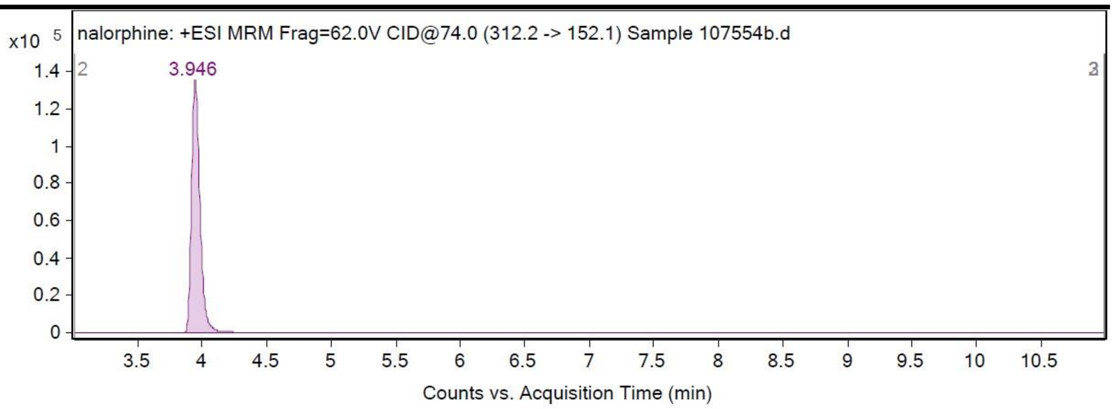

Integration Peak List

Peak	Start	RT	End	Height	Area	Area %
1	3.827	3.946	4.681	131164	629384	100

--- End Of Report ---

Appendix 4: MRM Sample 107559

Qualitative Analysis Report

**Data File**	Sample 107559b.d	**Sample Name**	Sample 107559b
**Sample Type**	Sample	**Position**	P2-D5
**Instrument Name**	Instrument 1	**User Name**	
**Acq Method**	Mitragynine_LMJ_Flexi_2.m	**Acquired Time**	31/10/2020 8:22:00 AM (UTC+08:00)
**IRM Calibration Status**	Not Applicable	**DA Method**	APCI Chemstation.m
**Comment**			
**Sample Group**		**Info.**	
**Stream Name**	LC 1	**Acquisition Time (Local)**	31/10/2020 8:22:00 AM (UTC+08:00)
**Acquisition SW Version**	6400 Series Triple Quadrupole B.08.02 (B8260.0)		

Chromatograms

**Fragmentor Voltage**	176	**Collision Energy**	50	**Ionization Mode**	ESI

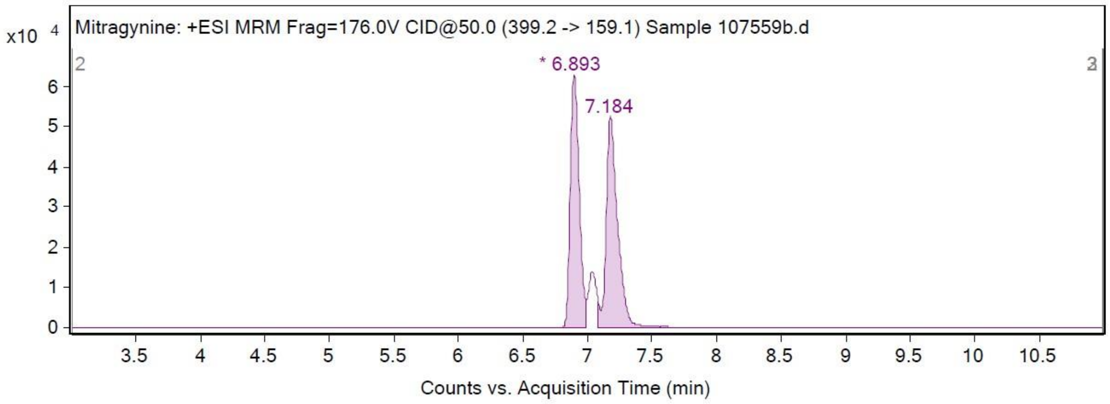

Integration Peak List

Peak	Start	RT	End	Height	Area	Area %
1	6.313	6.893	6.992	63058	311960	95.78
2	7.086	7.184	7.984	49101	325721	100

**Fragmentor Voltage**	176	**Collision Energy**	29	**Ionization Mode**	ESI

Qualitative Analysis Report

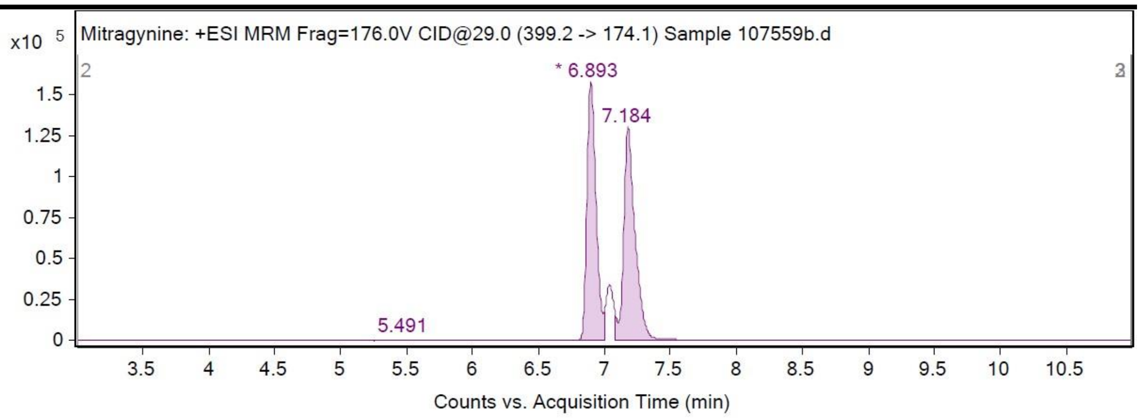

Integration Peak List

Peak	Start	RT	End	Height	Area	Area %
1	5.253	5.491	5.822	22	257	0.03
2	6.583	6.893	6.996	158123	768454	97.01
3	7.086	7.184	8.254	121347	792174	100

Fragmentor Voltage	176	Collision Energy	29	Ionization Mode	ESI

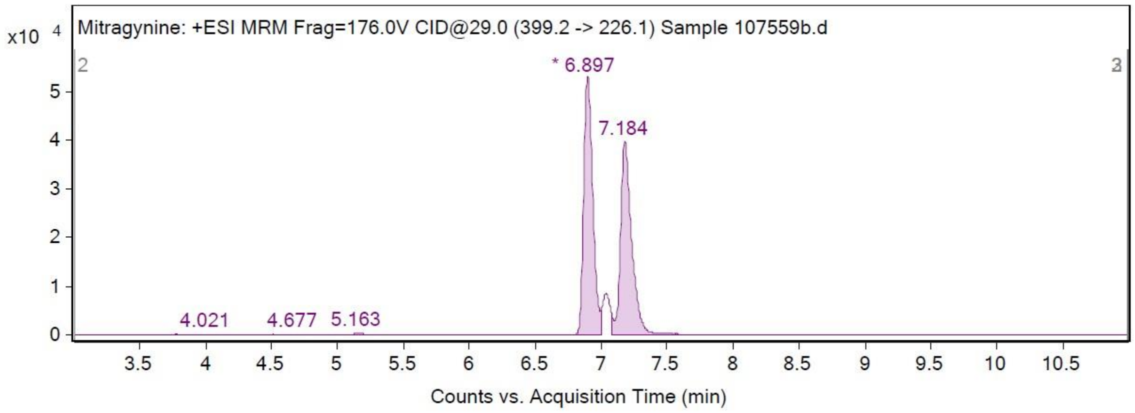

Integration Peak List

Peak	Start	RT	End	Height	Area	Area %
1	3.772	4.021	4.343	39	326	0.13
2	4.508	4.677	4.916	34	171	0.07
3	5.02	5.163	5.408	204	1239	0.48
4	6.774	6.897	6.996	53187	257663	100
5	7.081	7.184	7.872	37579	237844	92.31

Fragmentor Voltage	176	Collision Energy	21	Ionization Mode	ESI

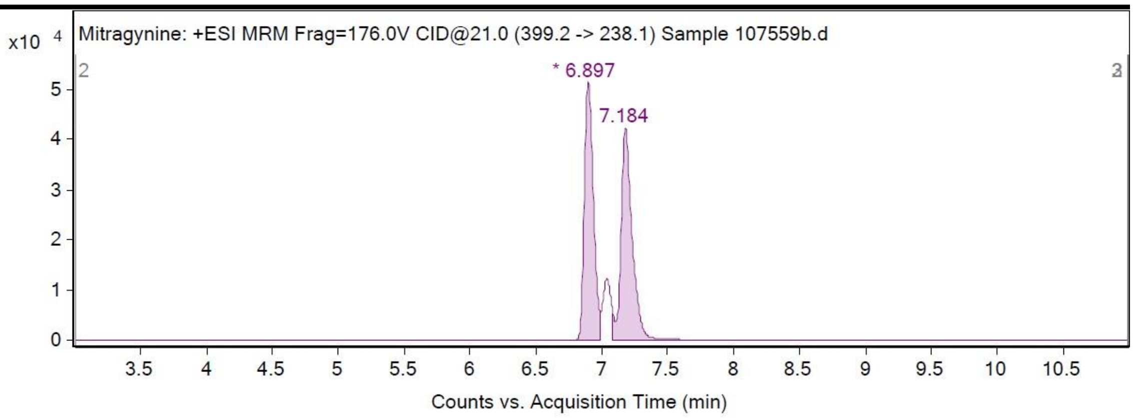

Integration Peak List

Peak	Start	RT	End	Height	Area	Area %
1	6.774	6.897	6.992	51503	250656	100
2	7.088	7.184	7.968	39938	250375	99.89

**Fragmentor Voltage**	6<br>2	**Collision Energy**	11<br>0	**Ionization Mode**	ES<br>I

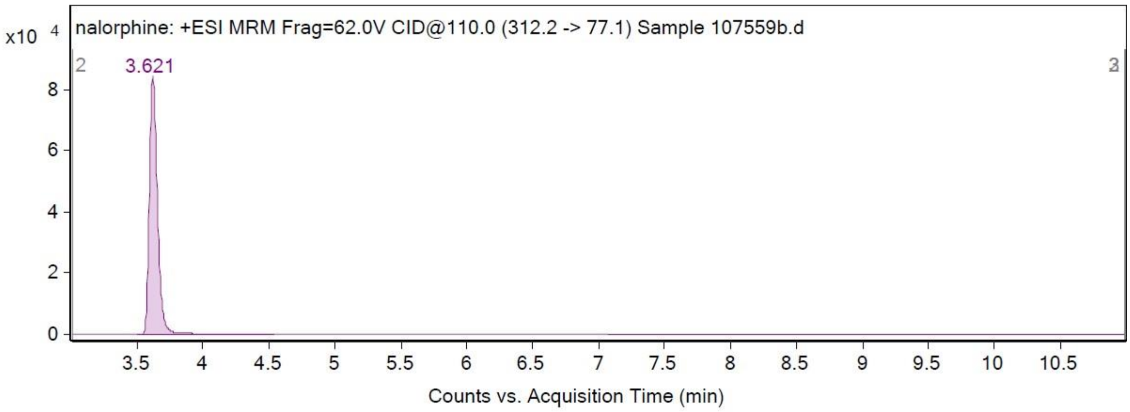

Integration Peak List

Peak	Start	RT	End	Height	Area	Area %
1	3.501	3.621	4.538	83842	366729	100

**Fragmentor Voltage**	6<br>2	**Collision Energy**	7<br>4	**Ionization Mode**	ES<br>I

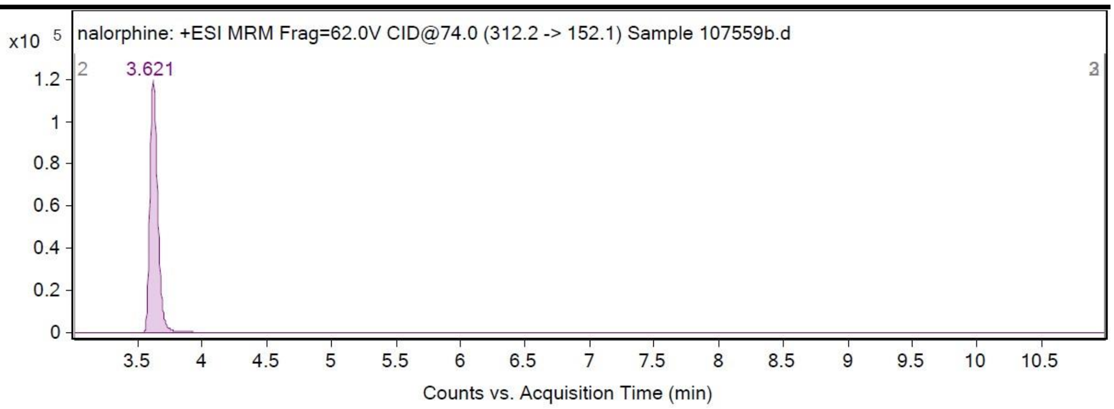

Integration Peak List

Peak	Start	RT	End	Height	Area	Area %
1	3.502	3.621	4.871	118818	517878	100

--- End Of Report ---

Appendix 5: MRM Sample 107561

Qualitative Analysis Report

**Data File**	Sample 107561b.d	**Sample Name**	Sample 107561b
**Sample Type**	Sample	**Position**	P2-D9
**Instrument Name**	Instrument 1	**User Name**	
**Acq Method**	Mitragynine_LMJ_Flexi_2.m	**Acquired Time**	31/10/2020 9:50:35 AM (UTC+08:00)
**IRM Calibration Status**	Not Applicable	**DA Method**	APCI Chemstation.m
**Comment**			
**Sample Group**		**Info.**	
**Stream Name**	LC 1	**Acquisition Time (Local)**	31/10/2020 9:50:35 AM (UTC+08:00)
**Acquisition SW Version**	6400 Series Triple Quadrupole B.08.02 (B8260.0)		

Chromatograms

**Fragmentor Voltage**	17<br>6	**Collision Energy**	5<br>0	**Ionization Mode**	ES<br>I

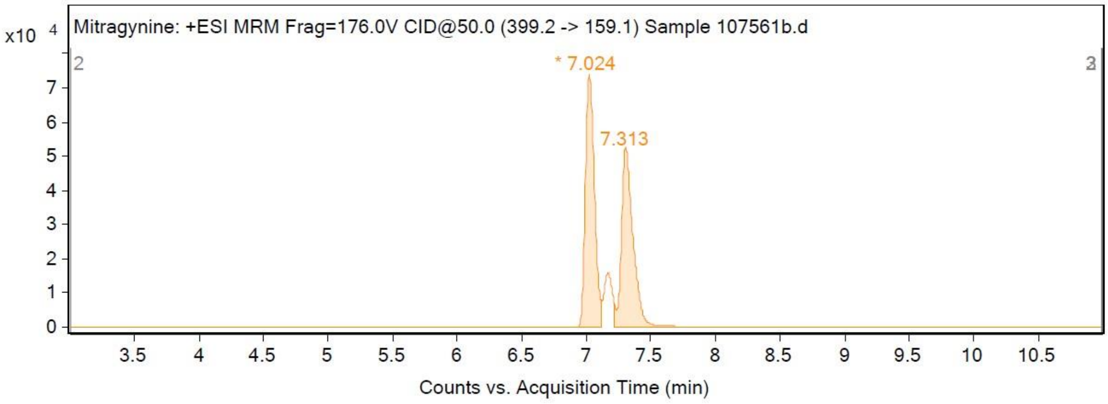

Integration Peak List

Peak	Start	RT	End	Height	Area	Area %
1	6.885	7.024	7.115	74019	363612	100
2	7.215	7.313	8.095	49831	334445	91.98

**Fragmentor Voltage**	17<br>6	**Collision Energy**	2<br>9	**Ionization Mode**	ES<br>I

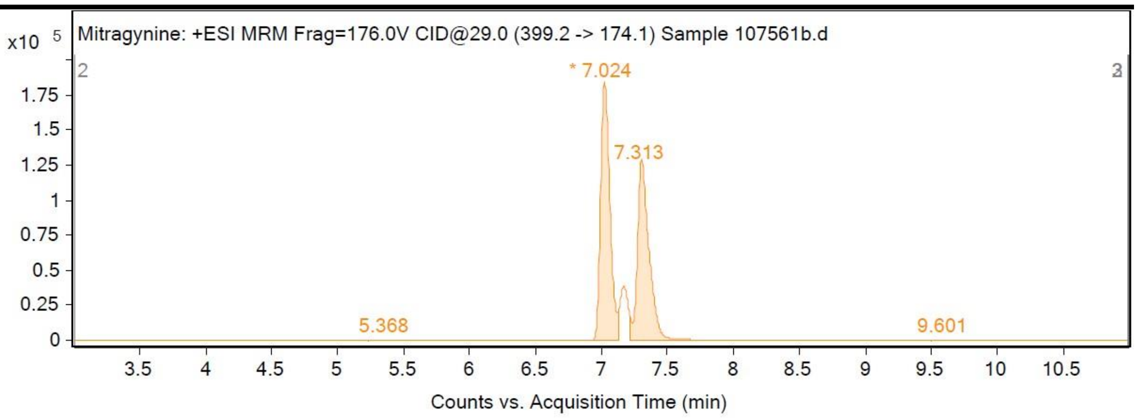

Integration Peak List

Peak	Start	RT	End	Height	Area	Area %
1	5.225	5.368	5.663	190	1099	0.12
2	6.734	7.024	7.123	184639	908487	100
3	7.216	7.313	8.238	121785	805803	88.7
4	9.496	9.601	9.891	25	276	0.03

**Fragmentor Voltage**	176	**Collision Energy**	29	**Ionization Mode**	ESI

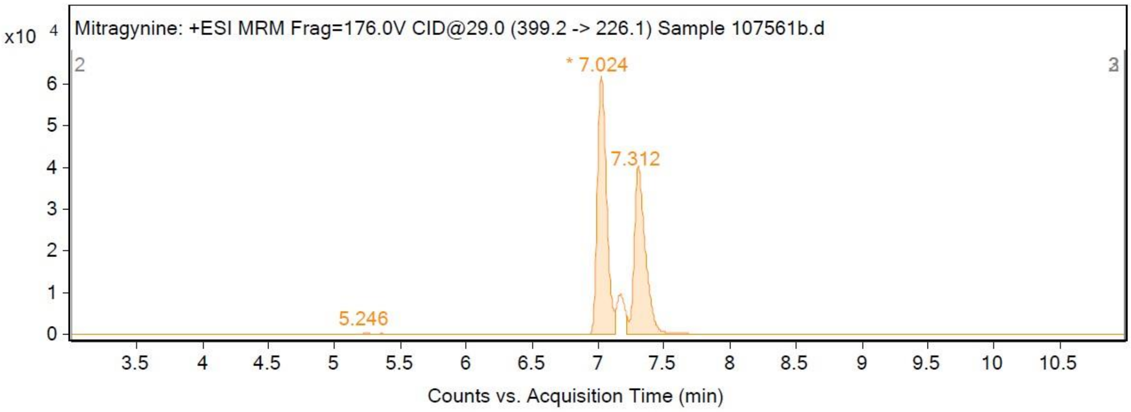

Integration Peak List

Peak	Start	RT	End	Height	Area	Area %
1	5.09	5.246	5.599	221	1892	0.63
2	6.905	7.024	7.123	61901	302595	100
3	7.213	7.312	8.063	37620	244159	80.69

**Fragmentor Voltage**	176	**Collision Energy**	21	**Ionization Mode**	ESI

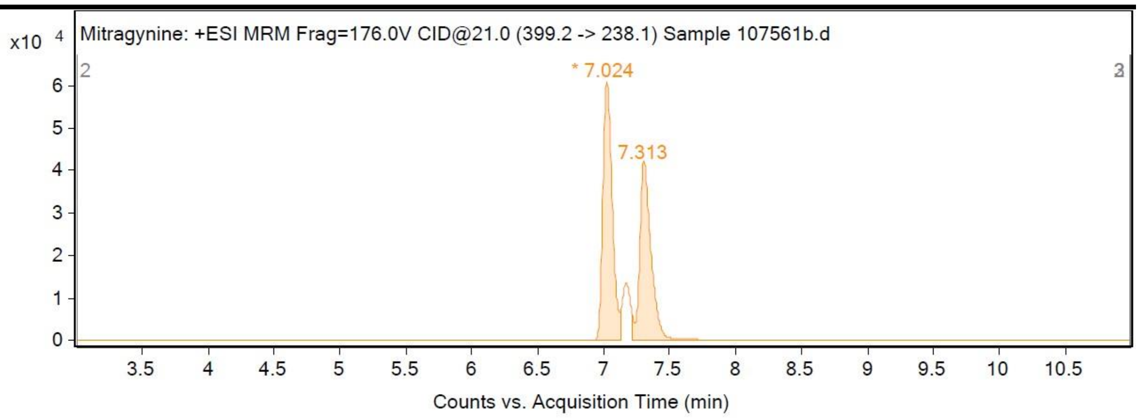

Integration Peak List

Peak	Start	RT	End	Height	Area	Area %
1	6.909	7.024	7.123	61050	302681	100
2	7.217	7.313	7.984	39980	255982	84.57

**Fragmentor Voltage**	6 2	**Collision Energy**	11 0	**Ionization Mode**	ES I

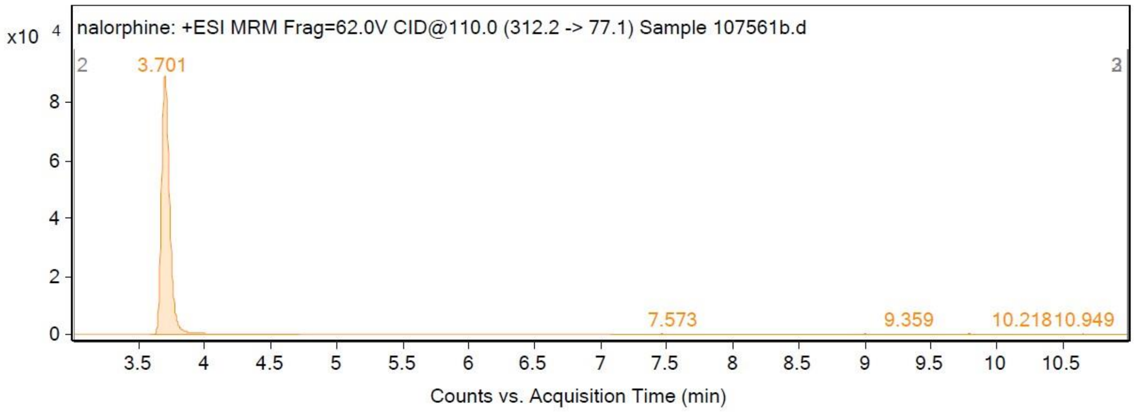

Integration Peak List

Peak	Start	RT	End	Height	Area	Area %
1	3.581	3.701	4.712	88486	389793	100
2	7.454	7.573	7.812	35	189	0.05
3	9.004	9.359	9.704	19	310	0.08
4	9.783	10.218	10.654	19	554	0.14
5	10.654	10.949	10.992	98	654	0.17

**Fragmentor Voltage**	6 2	**Collision Energy**	7 4	**Ionization Mode**	ES I

Qualitative Analysis Report

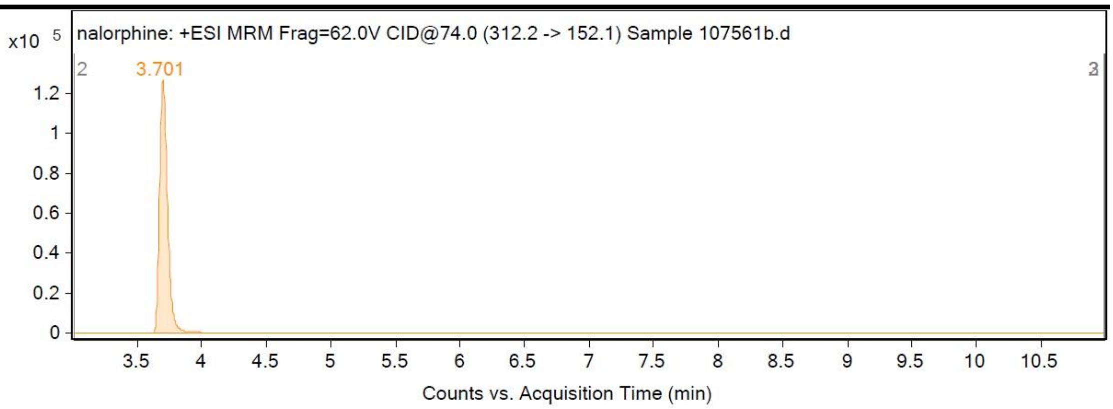

Integration Peak List

Peak	Start	RT	End	Height	Area	Area %
1	3.582	3.701	4.506	125409	552172	100

--- End Of Report ---

Appendix 6: MRM Sample 107563

Qualitative Analysis Report

Data File	Sample 107563a.d	Sample Name	Sample 107563a
Sample Type	Sample	Position	P2-E3
Instrument Name	Instrument 1	User Name	
Acq Method	Mitragynine_LMJ_Flexi_2.m	Acquired Time	31/10/2020 10:57:02 AM (UTC+08:00)
IRM Calibration Status	Not Applicable	DA Method	APCI Chemstation.m
Comment			
Sample Group		Info.	
Stream Name	LC 1	Acquisition Time (Local)	31/10/2020 10:57:02 AM (UTC+08:00)
Acquisition SW Version	6400 Series Triple Quadrupole B.08.02 (B8260.0)		

Chromatograms

Fragmentor Voltage	17<br>6	Collision Energy	5<br>0	Ionization Mode	ES<br>I

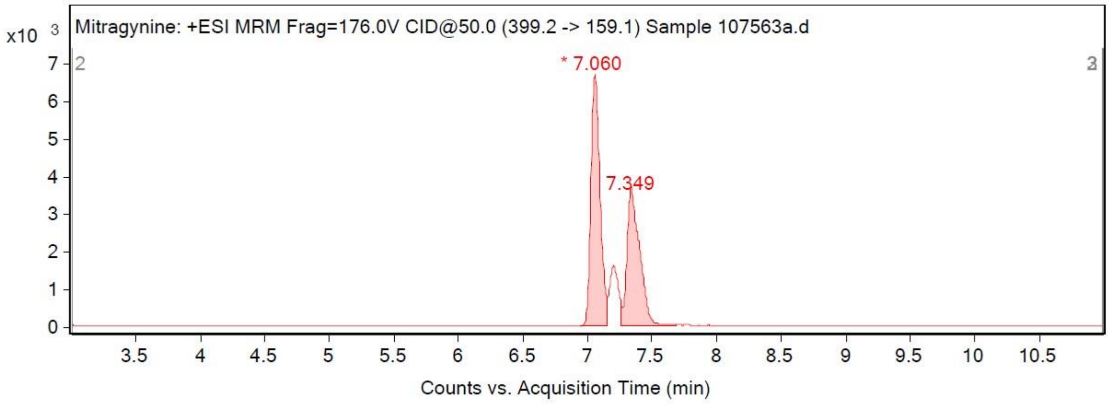

Integration Peak List

Peak	Start	RT	End	Height	Area	Area %
1	6.944	7.06	7.155	6685	32997	100
2	7.255	7.349	7.682	3475	25663	77.78

Fragmentor Voltage	17<br>6	Collision Energy	2<br>9	Ionization Mode	ES<br>I

Qualitative Analysis Report

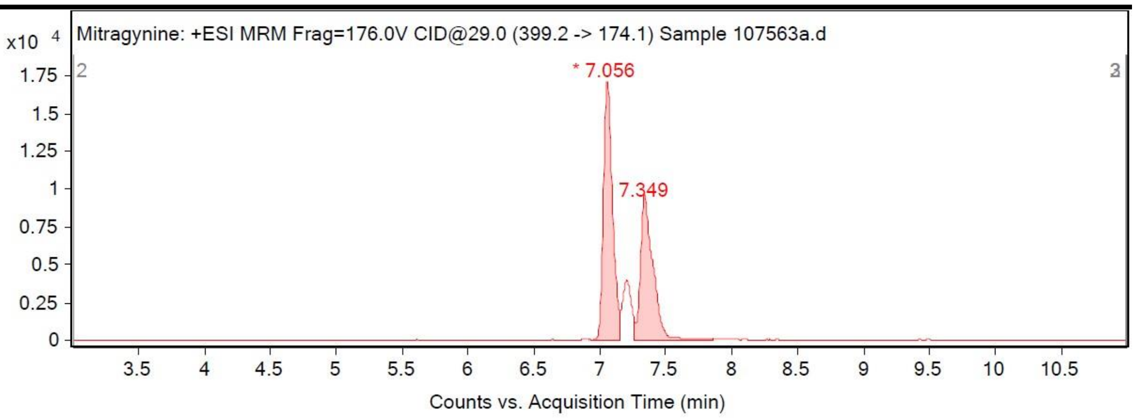

Integration Peak List

Peak	Start	RT	End	Height	Area	Area %
1	6.94	7.056	7.151	17104	82000	100
2	7.256	7.349	7.857	9092	63422	77.34

Fragmentor Voltage	176	Collision Energy	29	Ionization Mode	ESI

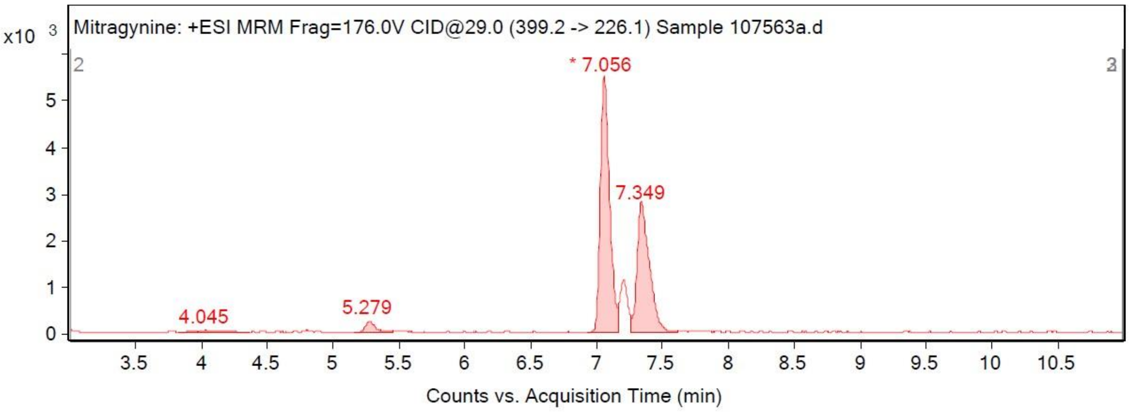

Integration Peak List

Peak	Start	RT	End	Height	Area	Area %
1	3.827	4.045	4.359	35	393	1.47
2	5.16	5.279	5.456	223	1123	4.2
3	6.925	7.056	7.159	5516	26756	100
4	7.253	7.349	7.618	2704	18382	68.7

Fragmentor Voltage	176	Collision Energy	21	Ionization Mode	ESI

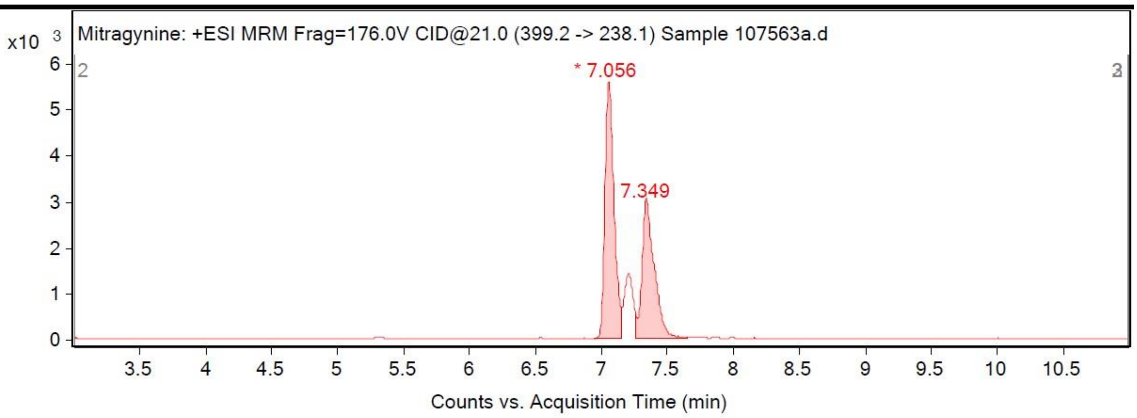

Integration Peak List

Peak	Start	RT	End	Height	Area	Area %
1	6.944	7.056	7.155	5589	26882	100
2	7.26	7.349	7.65	2916	19707	73.31

Fragmentor Voltage	6<br>2	Collision Energy	11<br>0	Ionization Mode	ES<br>I

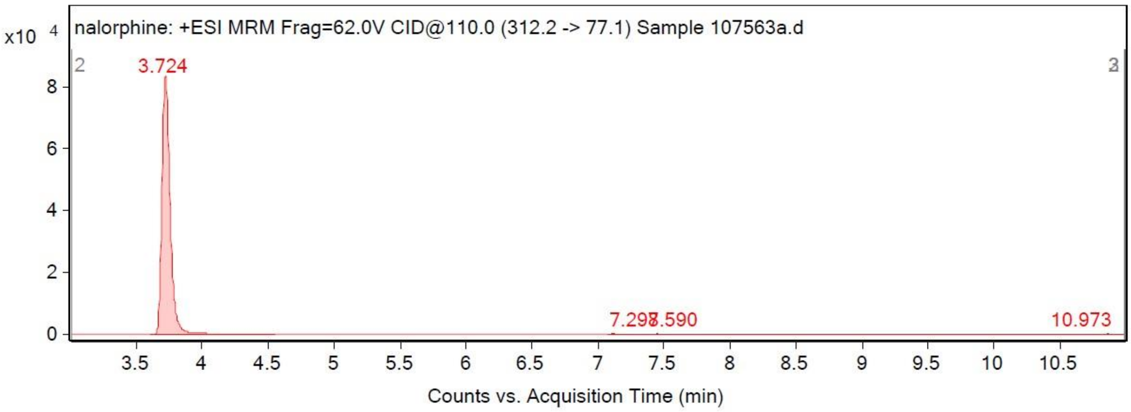

Integration Peak List

Peak	Start	RT	End	Height	Area	Area %
1	3.605	3.724	4.553	80338	367758	100
2	7.108	7.298	7.446	28	159	0.04
3	7.446	7.59	7.796	18	108	0.03
4	10.862	10.973	10.992	89	382	0.1

Fragmentor Voltage	6<br>2	Collision Energy	7<br>4	Ionization Mode	ES<br>I

Qualitative Analysis Report

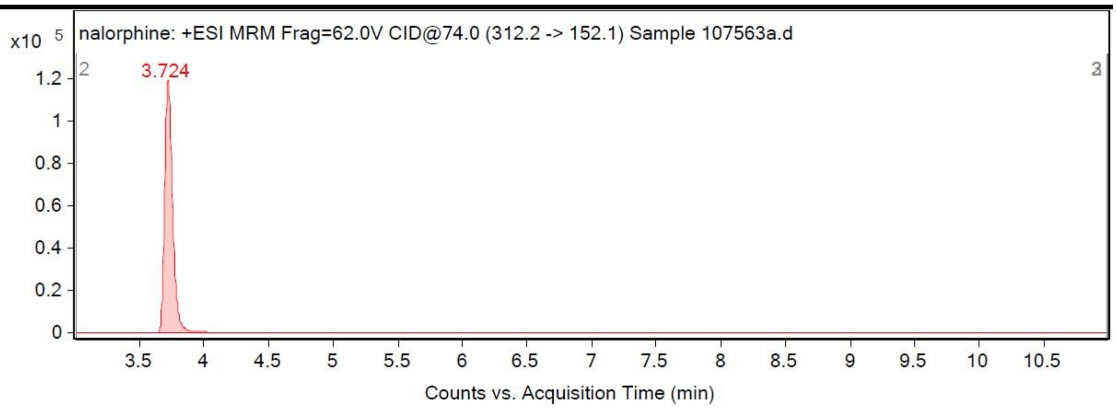

Integration Peak List

Peak	Start	RT	End	Height	Area	Area %
1	3.605	3.724	4.776	114617	522649	100

--- End Of Report ---

Appendix 7: MRM Sample 107564

Qualitative Analysis Report

**Data File**	Sample 107564a.d	**Sample Name**	Sample 107564a
**Sample Type**	Sample	**Position**	P2-E5
**Instrument Name**	Instrument 1	**User Name**	
**Acq Method**	Mitragynine_LMJ_Flexi_2.m	**Acquired Time**	31/10/2020 11:41:20 AM (UTC+08:00)
**IRM Calibration Status**	Not Applicable	**DA Method**	APCI Chemstation.m
**Comment**			
**Sample Group**		**Info.**	
**Stream Name**	LC 1	**Acquisition Time (Local)**	31/10/2020 11:41:20 AM (UTC+08:00)
**Acquisition SW Version**	6400 Series Triple Quadrupole B.08.02 (B8260.0)		

Chromatograms

**Fragmentor Voltage**	17 6	**Collision Energy**	5 0	**Ionization Mode**	ES I

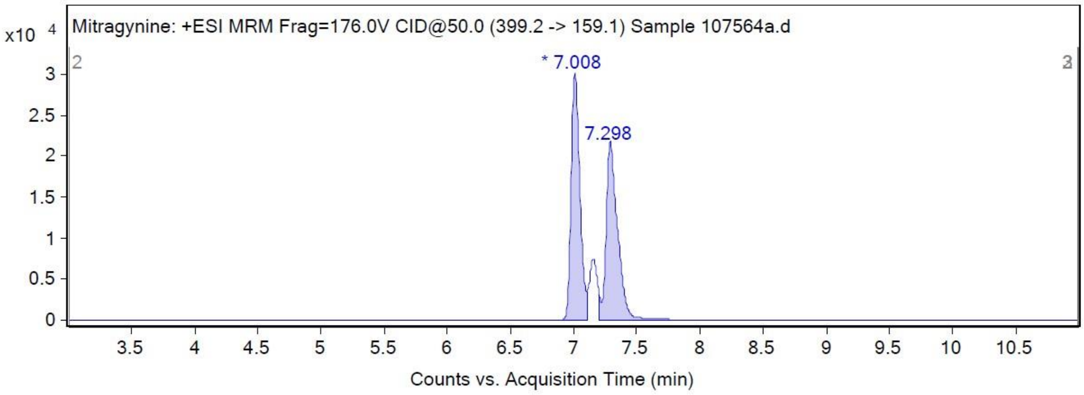

Integration Peak List

Peak	Start	RT	End	Height	Area	Area %
1	6.897	7.008	7.107	30241	146719	100
2	7.202	7.298	7.92	20530	138792	94.6

**Fragmentor Voltage**	17 6	**Collision Energy**	2 9	**Ionization Mode**	ES I

Qualitative Analysis Report

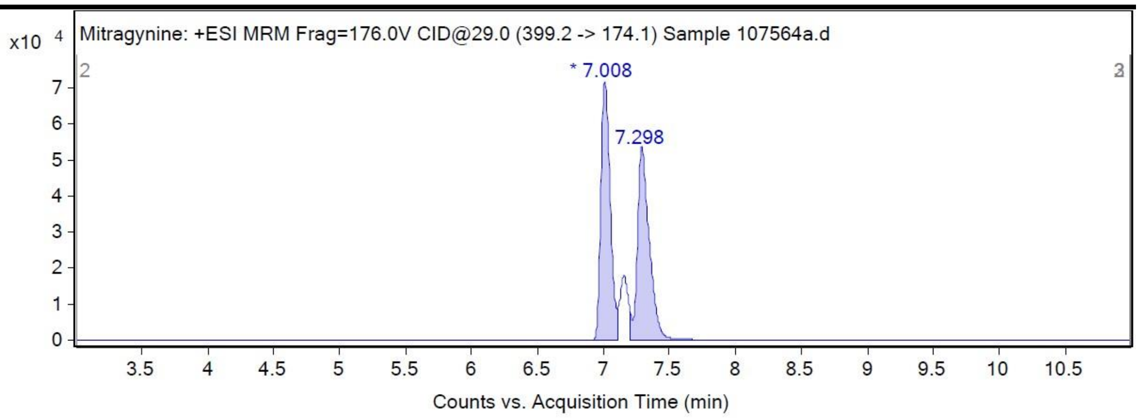

Integration Peak List

Peak	Start	RT	End	Height	Area	Area %
1	6.396	7.008	7.103	71866	358446	100
2	7.202	7.298	8.015	51226	343612	95.86

Fragmentor Voltage	17 6	Collision Energy	2 9	Ionization Mode	ES I

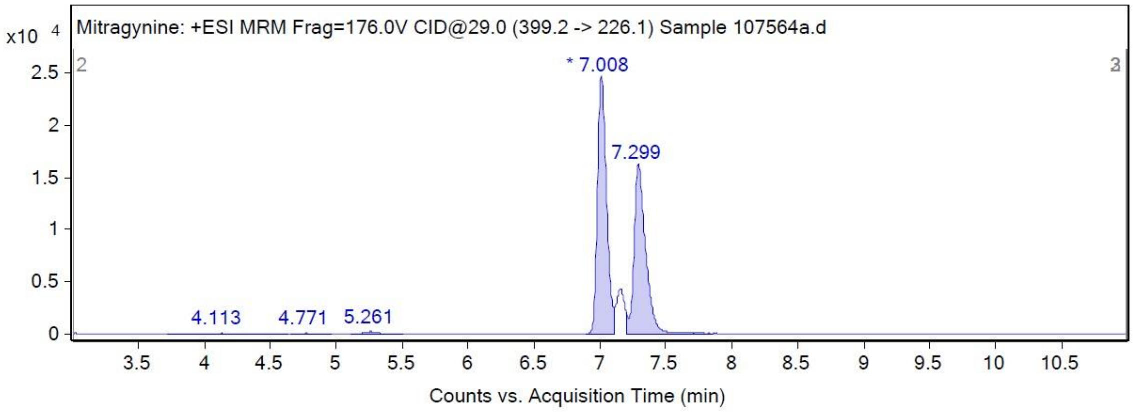

Integration Peak List

Peak	Start	RT	End	Height	Area	Area %
1	3.724	4.113	4.63	36	593	0.49
2	4.647	4.771	4.963	39	190	0.16
3	5.118	5.261	5.504	190	1203	1
4	6.897	7.008	7.111	24684	119906	100
5	7.199	7.299	7.857	15673	100546	83.85

Fragmentor Voltage	17 6	Collision Energy	2 1	Ionization Mode	ES I

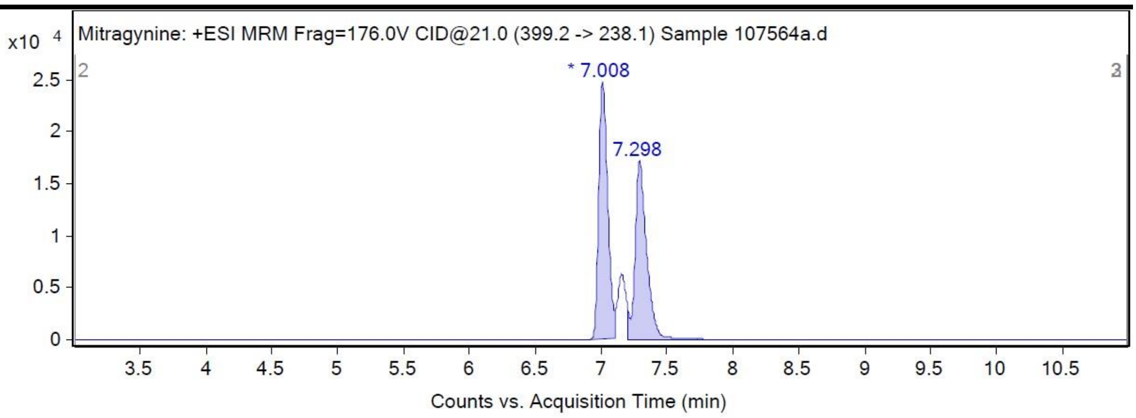

Integration Peak List

Peak	Start	RT	End	Height	Area	Area %
1	6.893	7.008	7.103	24787	118853	100
2	7.204	7.298	7.857	16505	105708	88.94

Fragmentor Voltage	6 2	Collision Energy	11 0	Ionization Mode	ES I

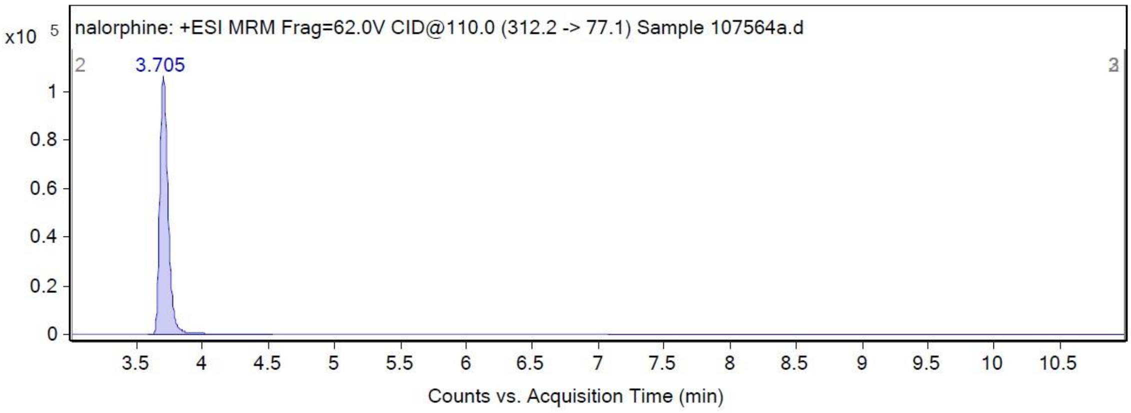

Integration Peak List

Peak	Start	RT	End	Height	Area	Area %
1	3.586	3.705	4.522	99857	473313	100

Fragmentor Voltage	6 2	Collision Energy	7 4	Ionization Mode	ES I

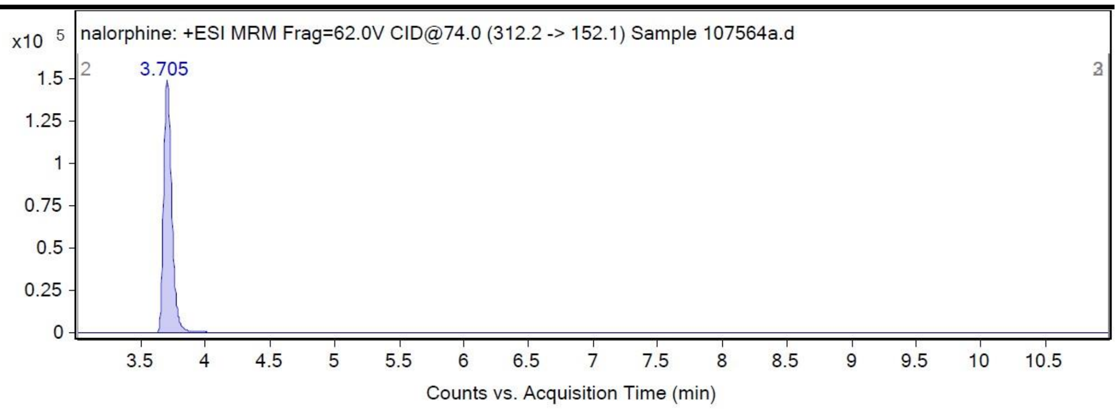

Integration Peak List

Peak	Start	RT	End	Height	Area	Area %
1	3.586	3.705	4.363	140540	666446	100

--- End Of Report ---

Appendix 8: MRM Sample 107565

Qualitative Analysis Report

**Data File**	Sample 107565a.d	**Sample Name**	Sample 107565a
**Sample Type**	Sample	**Position**	P2-E7
**Instrument Name**	Instrument 1	**User Name**	
**Acq Method**	Mitragynine_LMJ_Flexi_2.m	**Acquired Time**	31/10/2020 12:25:35 PM (UTC+08:00)
**IRM Calibration Status**	Not Applicable	**DA Method**	APCI Chemstation.m
**Comment**			
**Sample Group**		**Info.**	
**Stream Name**	LC 1	**Acquisition Time (Local)**	31/10/2020 12:25:35 PM (UTC+08:00)
**Acquisition SW Version**	6400 Series Triple Quadrupole B.08.02 (B8260.0)		

Chromatograms

**Fragmentor Voltage**	176	**Collision Energy**	50	**Ionization Mode**	ESI

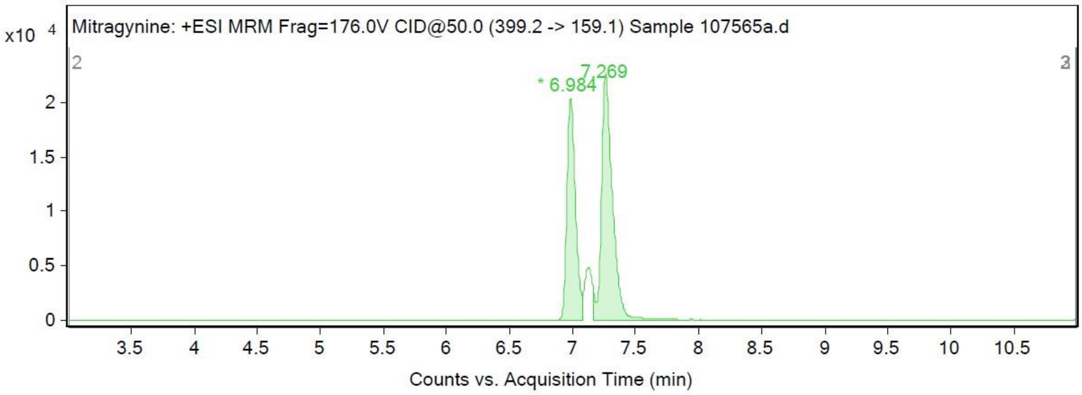

Integration Peak List

Peak	Start	RT	End	Height	Area	Area %
1	6.869	6.984	7.084	20342	97318	70.82
2	7.167	7.269	7.904	21815	137418	100

**Fragmentor Voltage**	176	**Collision Energy**	29	**Ionization Mode**	ESI

Qualitative Analysis Report

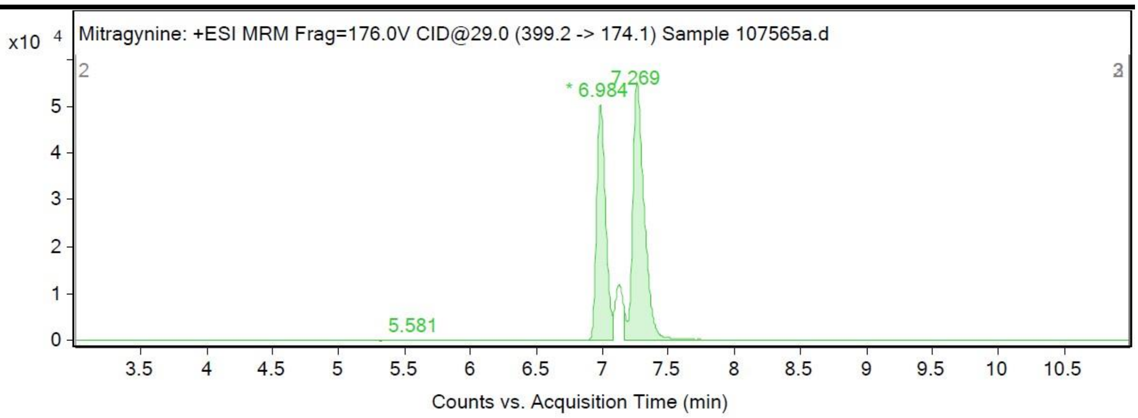

Integration Peak List

Peak	Start	RT	End	Height	Area	Area %
1	5.313	5.581	5.901	47	594	0.17
2	6.774	6.984	7.084	50283	242210	71.3
3	7.166	7.269	8.238	53460	339696	100

Fragmentor Voltage	176	Collision Energy	29	Ionization Mode	ESI

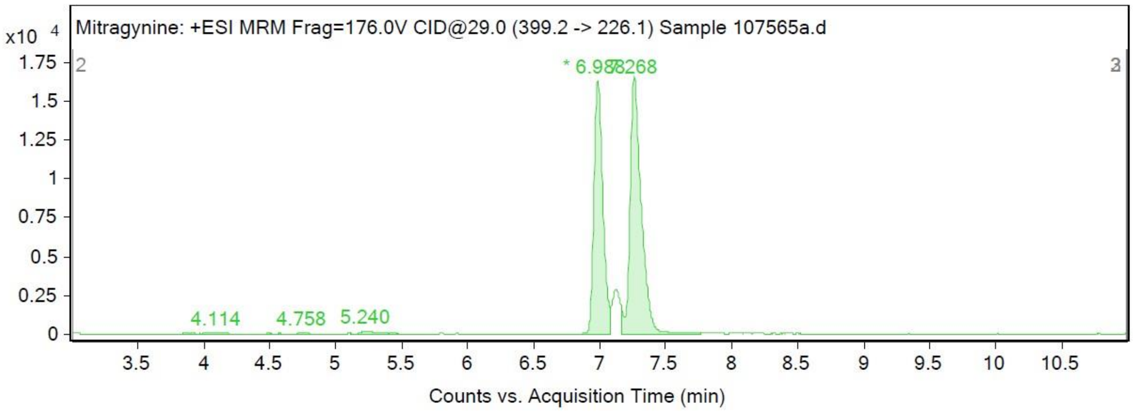

Integration Peak List

Peak	Start	RT	End	Height	Area	Area %
1	3.803	4.114	4.566	31	412	0.42
2	4.566	4.758	5.043	28	175	0.18
3	5.109	5.24	5.552	186	1265	1.29
4	6.869	6.988	7.08	16259	77082	78.45
5	7.163	7.268	7.761	15890	98258	100

Fragmentor Voltage	176	Collision Energy	21	Ionization Mode	ESI

Qualitative Analysis Report

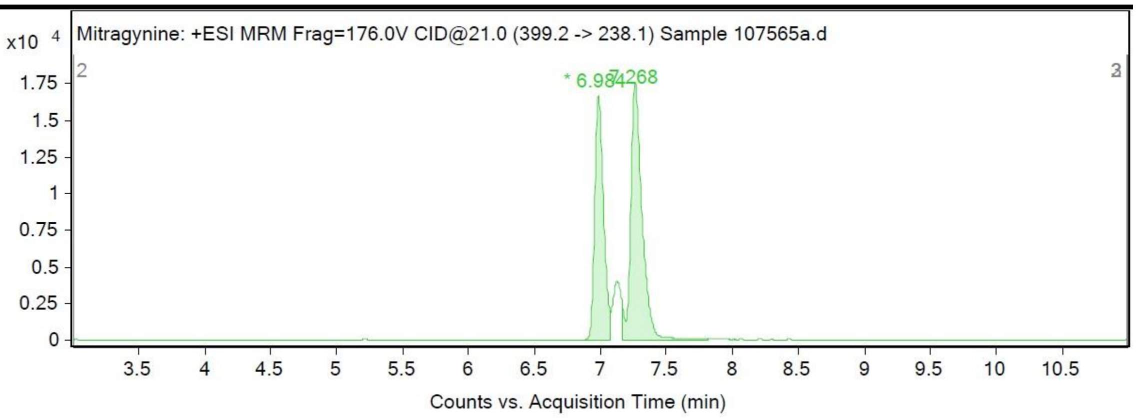

Integration Peak List

Peak	Start	RT	End	Height	Area	Area %
1	6.869	6.984	7.076	16612	78193	74.66
2	7.168	7.268	7.809	17058	104730	100

Fragmentor Voltage	62	Collision Energy	110	Ionization Mode	ESI

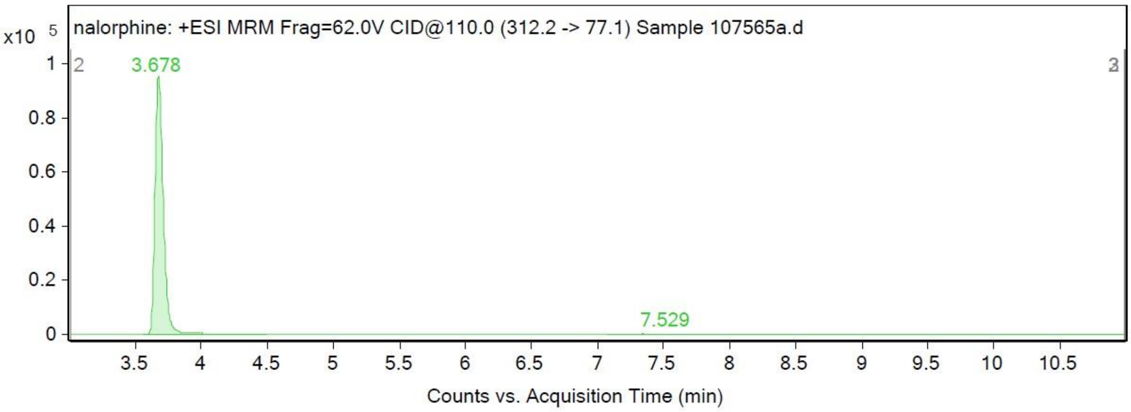

Integration Peak List

Peak	Start	RT	End	Height	Area	Area %
1	3.559	3.678	4.49	92736	421905	100
2	7.335	7.529	8.098	22	346	0.08

Fragmentor Voltage	62	Collision Energy	74	Ionization Mode	ESI

Qualitative Analysis Report

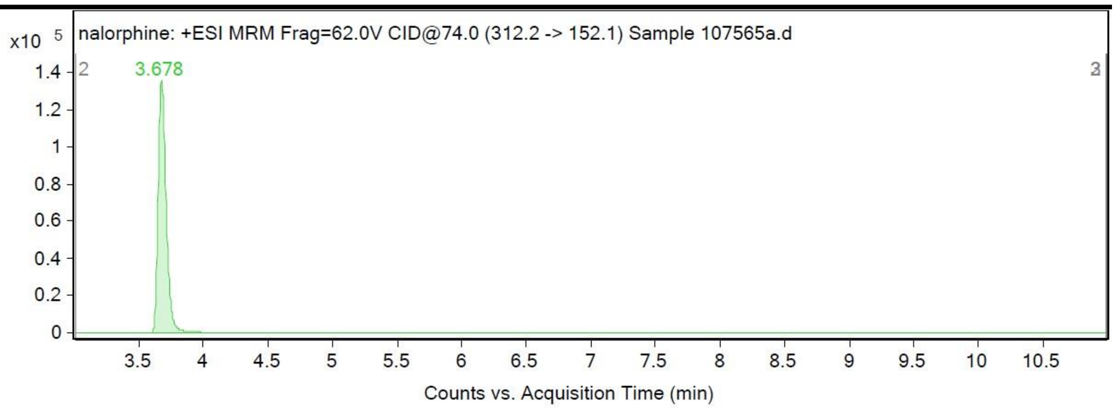

Integration Peak List

Peak	Start	RT	End	Height	Area	Area %
1	3.559	3.678	4.776	132230	596805	100

--- End Of Report ---

Appendix 9: MRM Sample 107566

Qualitative Analysis Report

**Data File**	Sample 107566b.d	**Sample Name**	Sample 107566b
**Sample Type**	Sample	**Position**	P2-F1
**Instrument Name**	Instrument 1	**User Name**	
**Acq Method**	Mitragynine_LMJ_Flexi_2.m	**Acquired Time**	31/10/2020 1:32:01 PM (UTC+08:00)
**IRM Calibration Status**	Not Applicable	**DA Method**	APCI Chemstation.m
**Comment**			
**Sample Group**		**Info.**	
**Stream Name**	LC 1	**Acquisition Time (Local)**	31/10/2020 1:32:01 PM (UTC+08:00)
**Acquisition SW Version**	6400 Series Triple Quadrupole B.08.02 (B8260.0)		

Chromatograms

**Fragmentor Voltage**	176	**Collision Energy**	50	**Ionization Mode**	ESI

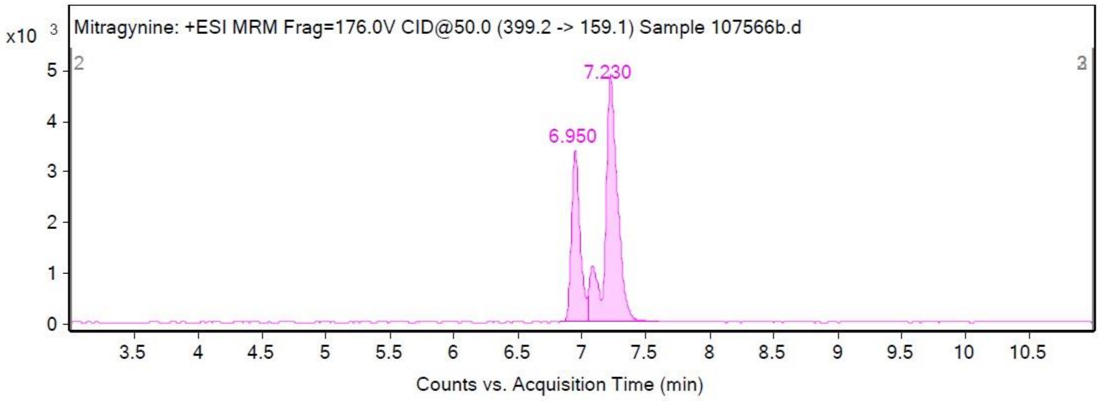

Integration Peak List

Peak	Start	RT	End	Height	Area	Area %
1	6.831	6.95	7.045	3334	16064	46.54
2	7.045	7.23	7.602	4427	34512	100

**Fragmentor Voltage**	176	**Collision Energy**	29	**Ionization Mode**	ESI

Qualitative Analysis Report

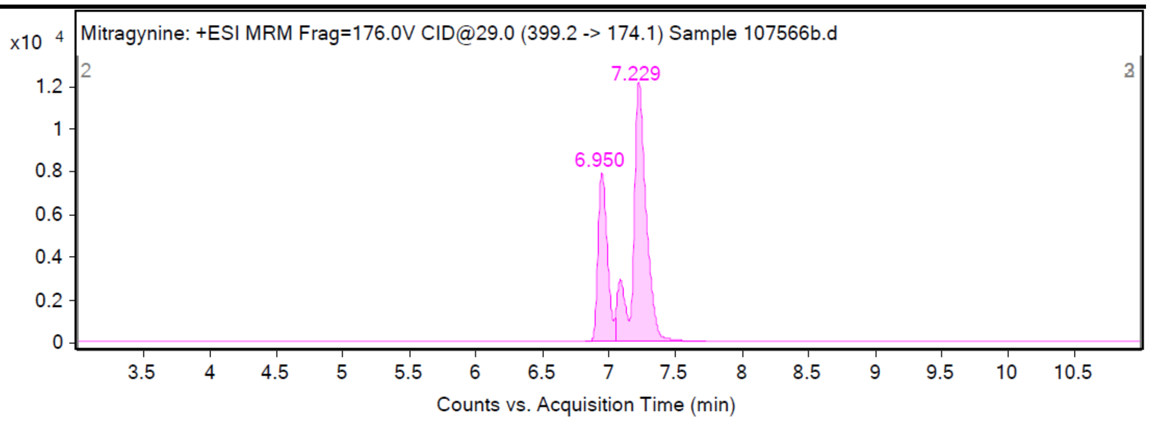

Integration Peak List

Peak	Start	RT	End	Height	Area	Area %
1	6.823	6.95	7.044	7769	39247	44.85
2	7.044	7.229	7.713	11292	87516	100

Fragmentor Voltage	17<br>6	Collision Energy	2<br>9	Ionization Mode	ES<br>I

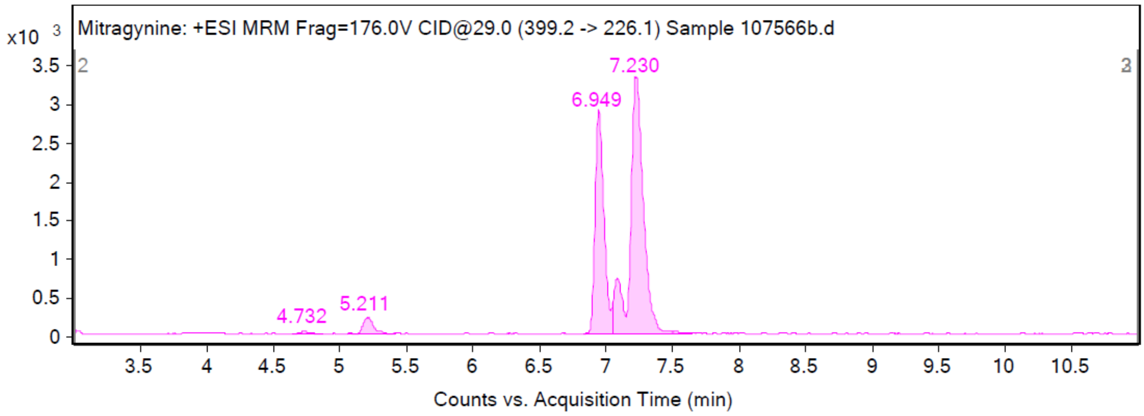

Integration Peak List

Peak	Start	RT	End	Height	Area	Area %
1	4.636	4.732	4.868	34	142	0.6
2	5.068	5.211	5.424	204	1156	4.84
3	6.83	6.949	7.05	2772	13542	56.73
4	7.05	7.23	7.65	3202	23870	100

Fragmentor Voltage	17<br>6	Collision Energy	2<br>1	Ionization Mode	ES<br>I

Qualitative Analysis Report

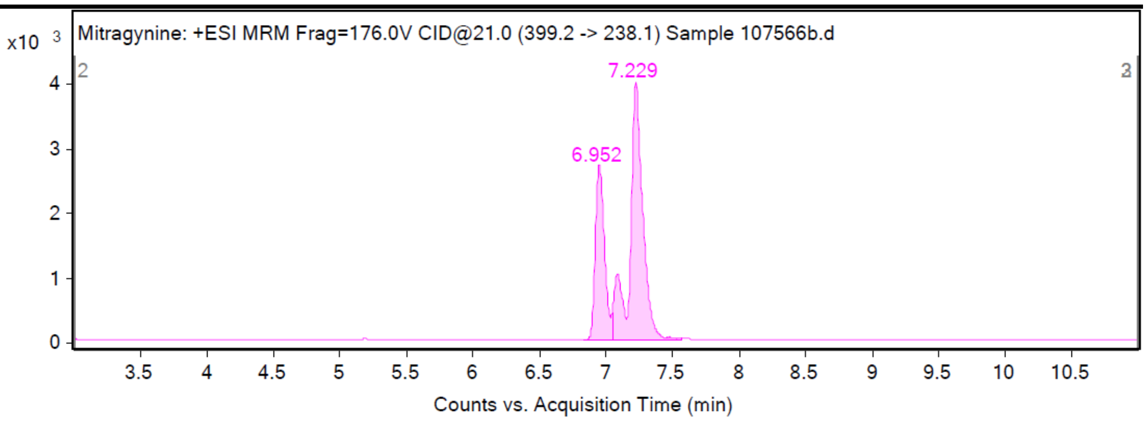

Integration Peak List

Peak	Start	RT	End	Height	Area	Area %
1	6.833	6.952	7.043	2657	13113	47.14
2	7.043	7.229	7.57	4009	27819	100

**Fragmentor Voltage**	62	**Collision Energy**	110	**Ionization Mode**	ESI

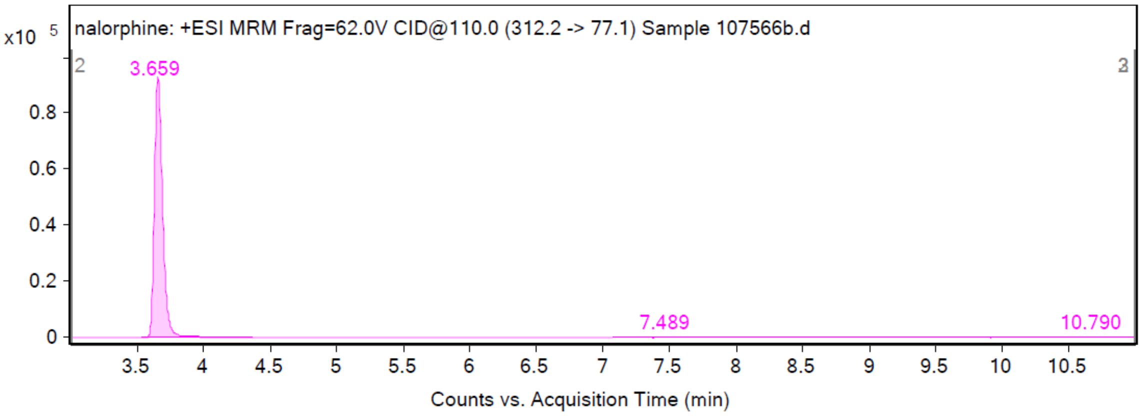

Integration Peak List

Peak	Start	RT	End	Height	Area	Area %
1	3.54	3.659	4.363	88718	405414	100
2	7.37	7.489	7.653	28	186	0.05
3	9.911	10.79	10.927	83	327	0.08

**Fragmentor Voltage**	62	**Collision Energy**	74	**Ionization Mode**	ESI

Qualitative Analysis Report

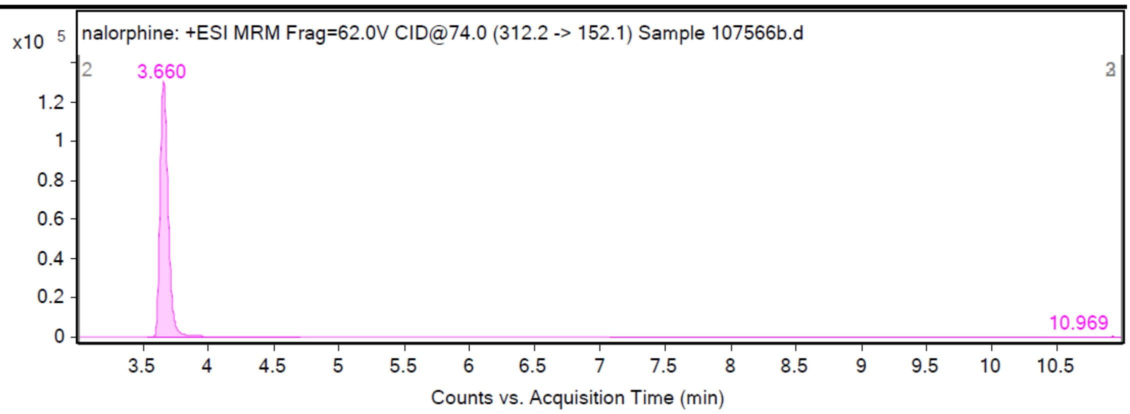

Integration Peak List

Peak	Start	RT	End	Height	Area	Area %
1	3.541	3.66	4.697	125451	575859	100
2	10.921	10.969	10.992	47	143	0.02

--- End Of Report ---

Appendix 10: MRM Sample 107567

Qualitative Analysis Report

**Data File**	Sample 107567a.d	**Sample Name**	Sample 107567a
**Sample Type**	Sample	**Position**	P2-F2
**Instrument Name**	Instrument 1	**User Name**	
**Acq Method**	Mitragynine_LMJ_Flexi_2.m	**Acquired Time**	31/10/2020 1:54:10 PM (UTC+08:00)
**IRM Calibration Status**	Not Applicable	**DA Method**	APCI Chemstation.m
**Comment**			
**Sample Group**		**Info.**	
**Stream Name**	LC 1	**Acquisition Time (Local)**	31/10/2020 1:54:10 PM (UTC+08:00)
**Acquisition SW Version**	6400 Series Triple Quadrupole B.08.02 (B8260.0)		

Chromatograms

**Fragmentor Voltage**	176	**Collision Energy**	50	**Ionization Mode**	ESI

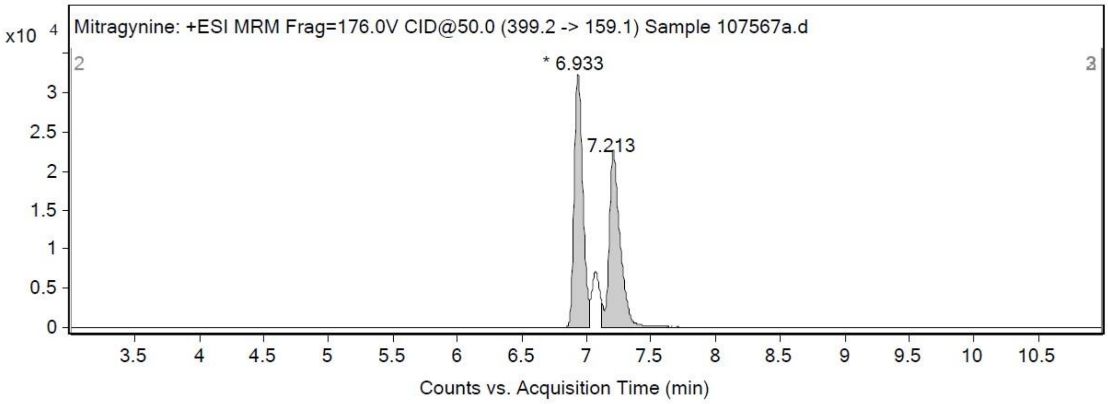

Integration Peak List

Peak	Start	RT	End	Height	Area	Area %
1	6.793	6.933	7.02	32356	151585	100
2	7.119	7.213	7.872	20483	135530	89.41

**Fragmentor Voltage**	176	**Collision Energy**	29	**Ionization Mode**	ESI

Qualitative Analysis Report

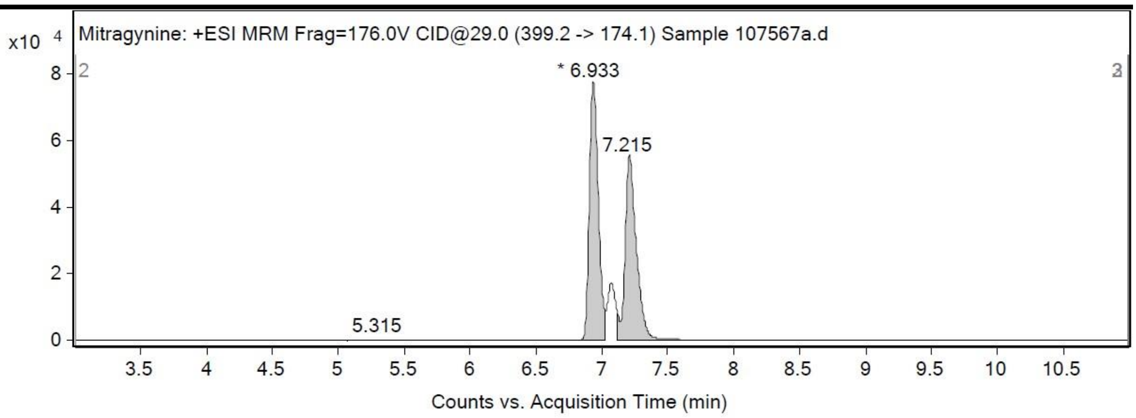

Integration Peak List

Peak	Start	RT	End	Height	Area	Area %
1	5.059	5.315	5.726	25	398	0.11
2	6.313	6.933	7.028	77849	372319	100
3	7.119	7.215	7.92	51916	335345	90.07

Fragmentor Voltage	17<br>6	Collision Energy	2<br>9	Ionization Mode	ES<br>I

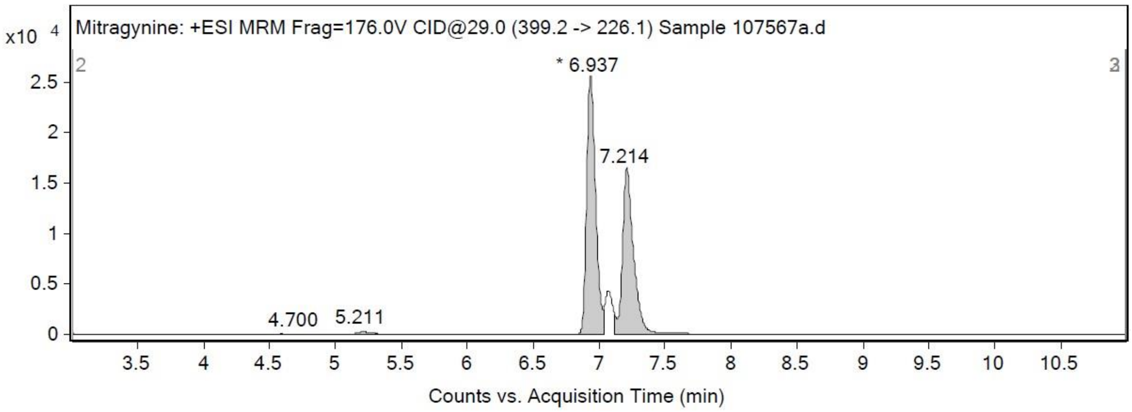

Integration Peak List

Peak	Start	RT	End	Height	Area	Area %
1	4.581	4.7	4.868	34	131	0.11
2	5.068	5.211	5.456	230	1355	1.11
3	6.817	6.937	7.032	25561	122006	100
4	7.116	7.214	7.745	15285	96939	79.45

Fragmentor Voltage	17<br>6	Collision Energy	2<br>1	Ionization Mode	ES<br>I

Qualitative Analysis Report

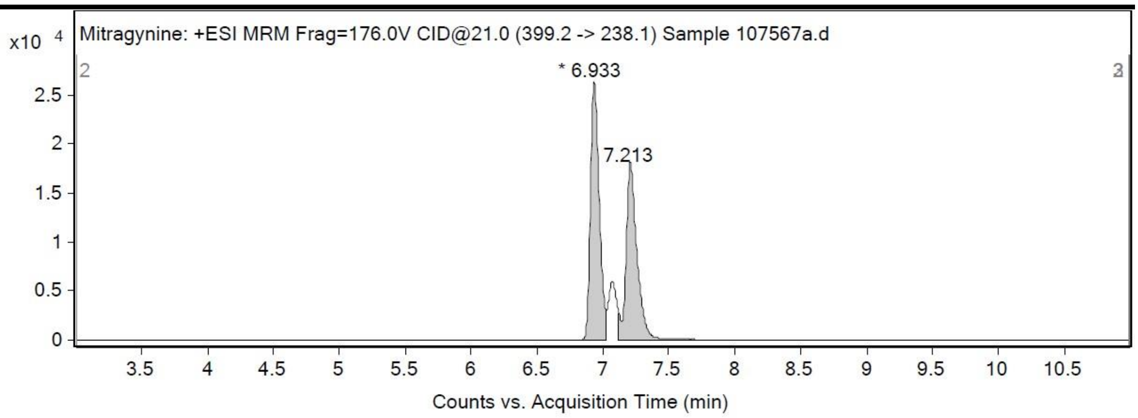

Integration Peak List

Peak	Start	RT	End	Height	Area	Area %
1	6.817	6.933	7.024	26377	123311	100
2	7.121	7.213	7.761	16515	104331	84.61

Fragmentor Voltage	6<br>2	Collision Energy	11<br>0	Ionization Mode	ES<br>I

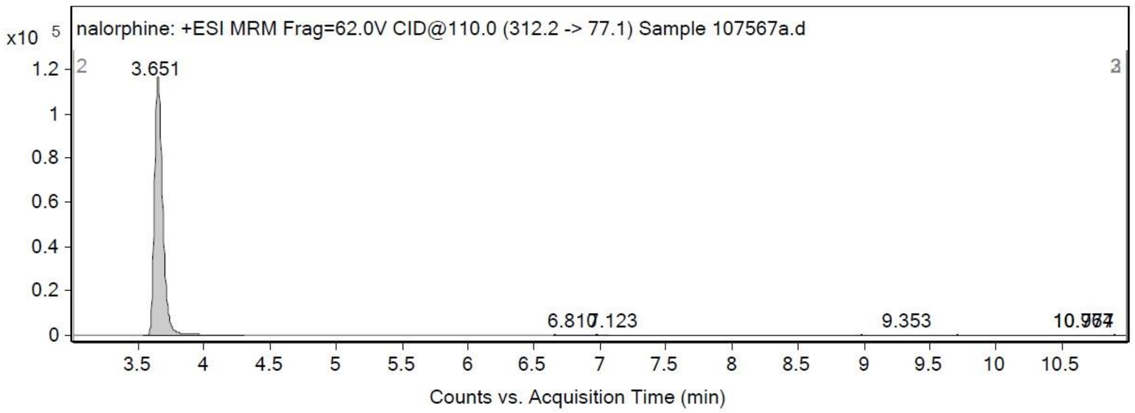

Integration Peak List

Peak	Start	RT	End	Height	Area	Area %
1	3.532	3.651	4.299	115694	514930	100
2	6.652	6.81	6.967	16	152	0.03
3	6.967	7.123	7.558	28	318	0.06
4	8.976	9.353	9.708	31	568	0.11
5	9.708	10.767	10.9	104	1667	0.32
6	10.9	10.974	10.992	59	222	0.04

Fragmentor Voltage	6<br>2	Collision Energy	7<br>4	Ionization Mode	ES<br>I

Qualitative Analysis Report

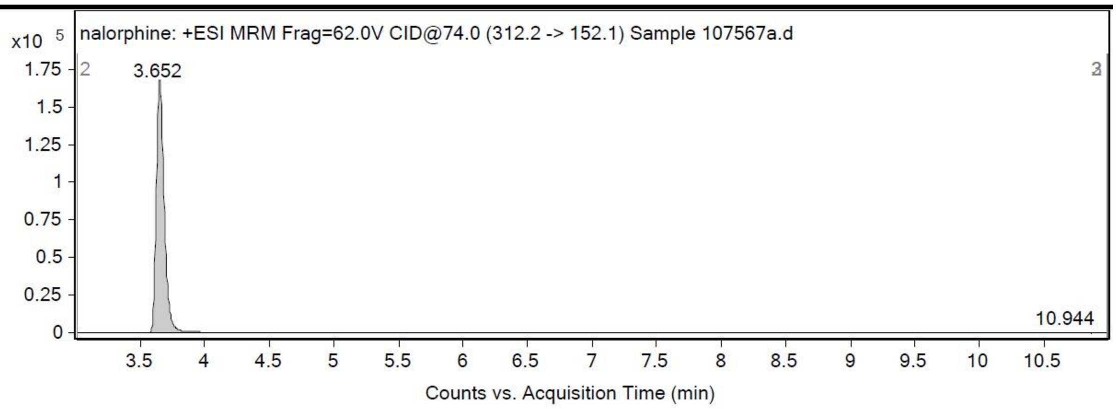

Integration Peak List

Peak	Start	RT	End	Height	Area	Area %
1	3.533	3.652	4.633	166673	736799	100
2	10.864	10.944	10.992	51	254	0.03

--- End Of Report ---
